# RET overexpression leads to increased brain metastatic competency in luminal breast cancer

**DOI:** 10.1093/jnci/djae091

**Published:** 2024-06-10

**Authors:** Petra Jagust, Aoibhin M Powell, Mihaela Ola, Louise Watson, Ana de Pablos-Aragoneses, Pedro García- Gómez, Ramón Fallon, Fiona Bane, Mona Heiland, Gareth Morris, Brenton Cavanagh, Jason McGrath, Daniela Ottaviani, Aisling Hegarty, Sinéad Cocchiglia, Kieron J Sweeney, Stephen MacNally, Francesca M Brett, Jane Cryan, Alan Beausang, Patrick Morris, Manuel Valiente, Arnold D K Hill, Damir Varešlija, Leonie S Young

**Affiliations:** Department of Surgery, RCSI University of Medicine and Health Sciences, Dublin, Ireland; School of Pharmacy and Biomolecular Sciences, RCSI University of Medicine and Health Sciences, Dublin, Ireland; Department of Surgery, RCSI University of Medicine and Health Sciences, Dublin, Ireland; Department of Surgery, RCSI University of Medicine and Health Sciences, Dublin, Ireland; Spanish National Cancer Research Center, Madrid, Spain; Spanish National Cancer Research Center, Madrid, Spain; Department of Surgery, RCSI University of Medicine and Health Sciences, Dublin, Ireland; Department of Surgery, RCSI University of Medicine and Health Sciences, Dublin, Ireland; Department of Physiology and Medical Physics, RCSI University of Medicine and Health Sciences, Dublin, Ireland; Department of Physiology and Medical Physics, RCSI University of Medicine and Health Sciences, Dublin, Ireland; Department of Neuroscience, Physiology and Pharmacology, University College London, London, UK; Cellular and Molecular Imaging Core, RCSI University of Medicine and Health Sciences, Dublin, Ireland; Department of Surgery, RCSI University of Medicine and Health Sciences, Dublin, Ireland; Department of Surgery, RCSI University of Medicine and Health Sciences, Dublin, Ireland; Department of Surgery, RCSI University of Medicine and Health Sciences, Dublin, Ireland; Department of Surgery, RCSI University of Medicine and Health Sciences, Dublin, Ireland; National Centre of Neurosurgery, Beaumont Hospital, Dublin, Ireland; National Centre of Neurosurgery, Beaumont Hospital, Dublin, Ireland; National Centre of Neurosurgery, Beaumont Hospital, Dublin, Ireland; Department of Neuropathology, National Centre of Neurosurgery, Beaumont Hospital, Dublin, Ireland; Department of Neuropathology, National Centre of Neurosurgery, Beaumont Hospital, Dublin, Ireland; Beaumont RCSI Cancer Centre, Beaumont Hospital, Dublin, Ireland; Spanish National Cancer Research Center, Madrid, Spain; Department of Surgery, RCSI University of Medicine and Health Sciences, Dublin, Ireland; School of Pharmacy and Biomolecular Sciences, RCSI University of Medicine and Health Sciences, Dublin, Ireland; Beaumont RCSI Cancer Centre, Beaumont Hospital, Dublin, Ireland; Department of Surgery, RCSI University of Medicine and Health Sciences, Dublin, Ireland; Beaumont RCSI Cancer Centre, Beaumont Hospital, Dublin, Ireland

## Abstract

**Background:**

Breast cancer brain metastasis is a rising occurrence, necessitating a better understanding of the mechanisms involved for effective management. Breast cancer brain metastases diverge notably from the primary tumor, with gains in kinase and concomitant losses of steroid signaling observed. In this study, we explored the role of the kinase receptor RET in promoting breast cancer brain metastases and provide a rationale for targeting this receptor.

**Methods:**

RET expression was characterized in a cohort of patients with primary and brain metastatic tumors. RET functionality was assessed using pharmacological inhibition and gene silencing in patient-derived brain metastatic tumor explants and *in vivo* models, organoid models, and brain organotypic cultures. RNA sequencing was used to uncover novel brain metastatic relevant RET mechanisms of action.

**Results:**

A statistically significant enrichment of RET in brain metastases was observed in estrogen receptor–positive breast cancer, where it played a role in promoting cancer cell adhesion, survival, and outgrowth in the brain. *In vivo*, RET overexpression enhanced brain metastatic competency in patient-derived models. At a mechanistic level, RET overexpression was found to enhance the activation of gene programs involved in cell adhesion, requiring EGFR cooperation to deliver a pro–brain metastatic phenotype.

**Conclusion:**

Our results illustrate, for the first time, the role of RET in regulating colonization and outgrowth of breast cancer brain metastasis and provide data to support the use of RET inhibitors in the management strategy for patients with breast cancer brain metastases.

Breast cancer brain metastases are an aggressive form occurring in 10 - 30% of breast cancer patients (1). The prevalence of breast cancer brain metastasis is on the rise, and the unique biological and molecular features have not yet been exploited sufficiently to develop specific therapeutic approaches. Gene expression profiling in models of triple-negative breast cancer and HER2-positive brain-homing cell lines has identified several key factors involved in various breast cancer brain metastasis–related processes. Though their relevance in the largest breast cancer subtype, estrogen receptor positive, is unclear (2-5). The delayed onset of breast cancer brain metastasis in estrogen receptor–positive/luminal disease suggests, in part, that luminal tumor cells possess a distinct capability to adapt to the brain compared with the more aggressive estrogen receptor–negative tumor cells. Emerging data, including ours, indicate that luminal tumor cells have more profound transcriptional remodeling events (6), some of which can endow intrinsic traits, resulting in brain-metastatic competency. Research into the genomics of metastatic samples has revealed that few recurrent mutations are specific to metastasis, even when compared with primary tumors with estrogen receptor 1 (*ESR1)* mutations being a notable exception linked to endocrine resistance (7-10). As such, no DNA-level alterations are likely to offer a single mechanistic insight into how breast cancer cells acquire brain metastatic proficiency, highlighting the need for an alternative approach to understand the aggressiveness of brain metastatic disease.

The receptor tyrosine kinase Ret proto-oncogene (RET) is the signaling receptor for the brain-specific glial–derived neurotrophic factor (GDNF) (11), and it has been reported to be overexpressed at the transcriptomic and proteomic levels in patients with breast cancer brain metastases (12). Overexpression, fusion and mutations of RET have been identified as oncogenic drivers in multiple types of cancer but are still rare in breast cancer (13-16). Two mechanisms of RET activation have been described, binding to GDNF and its co-receptor GDNF family receptor alpha 1-4 (GFRA1-4), which leads to dimerization and phosphorylation, and overexpression, both of which promote cell growth and survival (17-23). The RET signaling pathway has been linked to endocrine resistance in various preclinical and primary tumor models where RET is a direct target for estrogen receptor (13,24-27). Given the independently reported role of RET in estrogen receptor–positive disease (13,28) and its potential role in breast cancer brain metastasis, we sought to characterize RET’s involvement in estrogen receptor–positive breast cancer brain metastasis.

In this study, in an extensive cohort of matched primary and brain metastatic tumors, we report RET as one of the top-ranked actionable kinases, specifically in estrogen receptor–positive tumors. We uncover the drivers that enable RET overexpressing metastatic cells to migrate to the brain and adapt to the brain environment, as well as to determine the mechanisms that facilitate this process. At a clinical level, we observe that RET associates with poor clinical outcome and demonstrate RET overexpression has a metastatic advantage in *in vivo* patient models of breast cancer brain metastasis. Mechanistically, we provide evidence that RET can activate specific pathways involved in cell adhesion in cooperation with epidermal growth factor receptor (EGFR) to deliver a pro–brain metastatic phenotype. Our findings provide novel insights into the role of RET in controlling the colonization and expansion of breast cancer brain metastasis that could inform approaches to manipulate the RET-signaling axis as a treatment in breast cancer brain metastasis.

## Methods

An expanded description of methods and materials used in this study is provided in the Supplementary Materials file (available online).

### Patient samples

Clinical samples were obtained from the clinical trial Breast Cancer Proteomics and Molecular Heterogeneity (ClinicalTrials.gov identifier NCT01840293). Informed and written consent was obtained before any clinical material was collected.

### Immunohistochemistry 

Immunohistochemistry against RET (Merck, Rahway, NJ; No. HPA008356; RRID: AB_1847232) was performed on a tissue microarray of primary breast cancer tumors (n = 820 patients) following a previously published protocol (29).

### Establishment of breast cancer brain metastatic organoids and organotypic cultures 

The organoid cultures (6,29,30) and the breast cancer brain metastatic T347 and LY2-Mets cell lines brain and liver organotypic cultures were established as previously described (31).

### *In vivo* study

To analyze breast cancer brain metastatic cell brain colonization, T347 RET overexpressing (T347-RET^+^) and T347 control (T347-Ctrl) cells were delivered through intracardiac injection into the mouse left ventricle (n = 7 mice per condition). The development of metastases was assessed both *in vivo* and *ex vivo* using bioluminescence imaging.

### RNA sequencing

RNA sequencing was performed on T347-Ctrl and T347-RET^+^ cells using the BGISEQ 500 platform (BGI Genomics, Hong Kong). Data were analyzed using R packages (R Foundation for Statistical Computing, Vienna, Austria).

### Three-dimensional cell viability assay

Organoid fragments and single cells (1 × 10^4^ cells) from dissociated organoids were seeded on Cultrex Reduced Growth Factor Basement Membrane Matrix, type 2 (Trevigen/Bio-Techne, Minneapolis, MN; No. 3533-001-02) in organoid media (6,32). Pharmacological agent LOXO-292 (selpercatinib, gifted from Loxo-Oncology, Stamford, CT; Selleckchem, No. S8782) was added on day 0, and cell viability was assayed after 7 days using the CellTiter-Glo Assay 3D kit as per manufacturers protocol (Promega, Madison, WI, No. G9681). Luminescence was read on a microplate reader (Victor3, PerkinElmer, Waltham, MA).

### Statistical analysis

Microsoft Excel (2016; RRID: SCR_01613) and GraphPad Prism, version 9.2.0, software (RRID: SCR_002798) were used for graphical presentation of the data and statistical analysis. Bioinformatics data were presented using R packages (https://cran.r-project.org/bin/windows/base/). For association analysis of categorical variables, the Fisher exact test was used (StataSE-64.exe, StataCorp, College Station, TX; RRID: SCR_012763). Statistical analysis was performed using 2-tailed tests and Pearson correlation test, as described in the “Results” section. *P *<  .5 was considered statistically significant.

## Results

### RET is a key player in estrogen receptor–positive breast cancer brain metastasis

**Figure 1.**
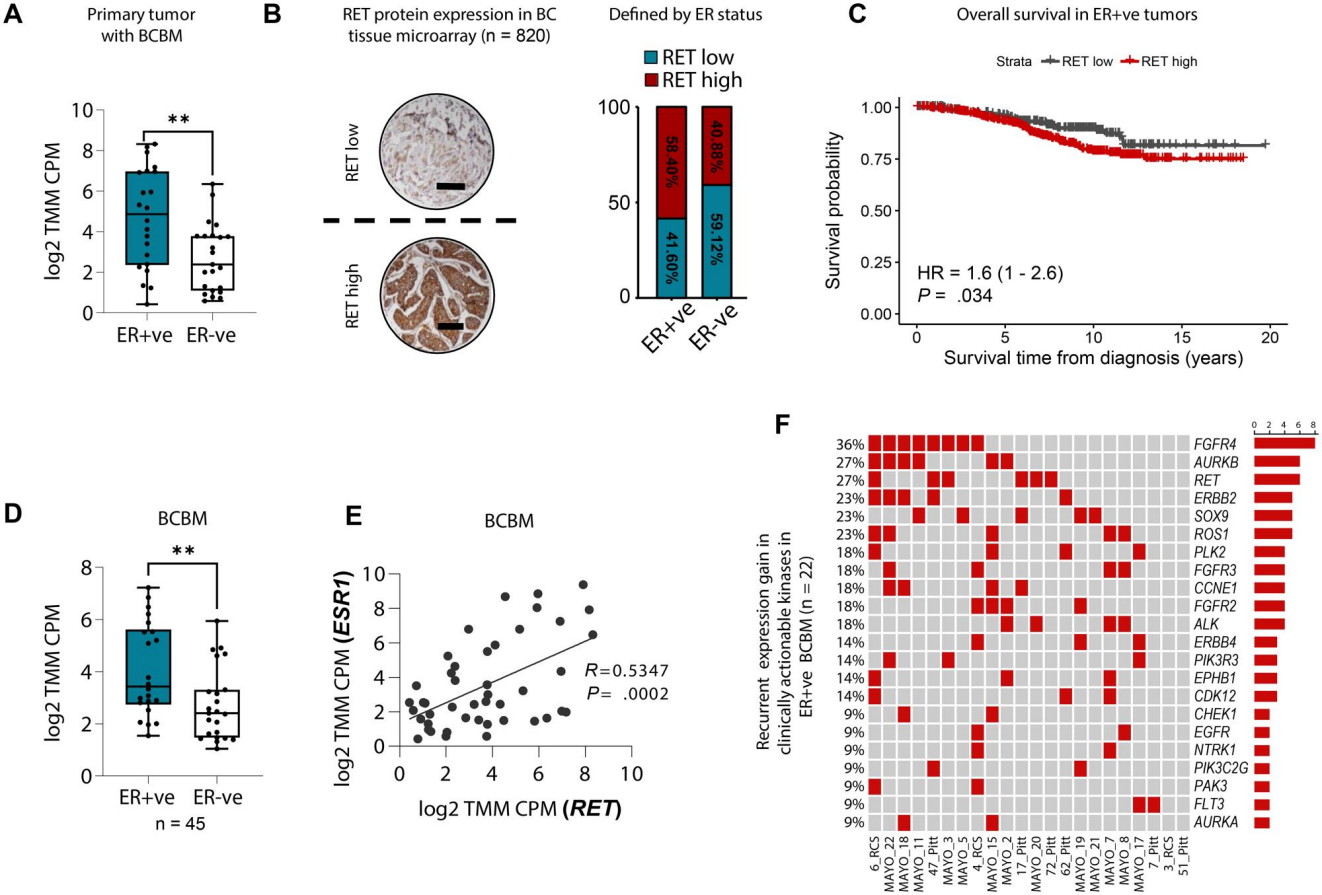
RET is a key player in estrogen receptor–positive breast cancer brain metastasis. **A**) *RET* gene expression based on estrogen receptor expression in primary tumors with BCBM (n = 45 patients). Whiskers go from the minimum to the maximum value. The *P* value was obtained using a 2-tailed *t* test. **P <* *.01. **B**) Representative images of immunohistochemical staining of RET protein on a tissue microarray (n = 820) of BC samples. Scale bars, 100 μm. **Dashed line** represents the cutoff for RET high and RET low expression samples. RET high and RET low cutoff (immunohistochemical cutoff score = 300) was obtained with the ROC curve (left). The percentage of RET high and RET low in the ER+ve (n = 661) and ER-ve (n = 159) patient population (right). **C**) Kaplan-Meier analysis of overall survival in ER+ve BC tissue (n = 661). **D**) *RET* gene expression in BCBM comparing ER-ve (n = 23) and ER+ve (n = 22) patient samples. The *P* value was obtained using a 2-tailed *t* test. **P <* *.01. **E**) Correlation of *ESR1* and *RET* gene expression (log2 TMM CPM) in BCBM patient samples (n = 45). The *P* value was obtained using a 2-tailed Pearson correlation test. **F**) OncoPrint of clinically actionable kinases with discrete expression gains in ER+ve BCBM patient samples (n = 45). BC = Breast Cancer; BCBM = Breast Cancer Brain Metastasis; ER+ve = Estrogen-Receptor positive; ER-ve = Estrogen-Receptor negative; ROC = Receiver Operating Characteristic curve.

In an extensive cohort of patients with breast cancer brain metastases (45 patients, 90 paired samples), we had previously identified RET as a key kinase elevated in metastasis (6). In this study, we found that *RET* messenger RNA was statistically significantly enriched in estrogen receptor–positive primary tumors that metastasize to the brain compared with estrogen receptor–negative tumors (Figure 1, A). Consistent with this, higher *RET* was also detected in estrogen receptor–positive primary tumors from the Molecular Taxonomy of Breast Cancer International Consortium cohort (33), independent of their breast cancer brain metastatic status (n = 1028) (Supplementary Figure 1, A, available online). We utilised a breast cancer tissue microarray to validate RET as a target and investigated its utility as a predictor of poor outcome and for the identification of high-risk patients. At the protein level, RET was found to be expressed in estrogen receptor–positive and estrogen receptor–negative breast primary tumors (Figure 1, B), where it is associated with poor overall survival in estrogen receptor–positive (hazard ratio [HR] = 1.6, *P* = .034) but not HER2-positive (*P* = .62) or triple-negative tumors (*P* = .76) (Figure 1, C; Supplementary Figure 1, B and C; Supplementary Table 1, available online). Similarly, in the Kaplan-Meier plotter (Bio-protocol, Sunnyvale, CA) (34), an association between elevated *RET* expression and poor recurrence-free survival and distant metastasis-free survival (HR = 1.26, *P* = .0071 and HR = 1.61, *P* = .0074, respectively) was observed (Supplementary Figure 1, D, available online).

In a metastatic cohort, we found *RET* enriched in estrogen receptor–positive breast cancer brain metastasis compared with estrogen receptor–negative breast cancer brain metastasis (Figure 1, D). Moreover, high *RET* gene expression positively correlates with the *ESR1* in breast cancer brain metastatic patient samples (Figure 1, E). Analysis of estrogen receptor–positive brain metastatic tumors with their matched primary breast tumor, highlighted *RET* as one of the top enriched clinically actionable kinase genes, with expression gains in 27% of cases (Figure 1, F). Aurora kinase B (*AURKB*) and fibroblast growth factor receptor 4 (*FGFR4*) were also highly enriched. While these genes make interesting targets due to their frequent overexpression in breast cancer metastases treated with endocrine therapy (7,35), the emerging interest in addressing wild-type *RET* in metastasis—coupled with our recent identification of RET overexpression in breast cancer brain metastasis—prompted a focused investigation into RET. Results here underscore the pivotal role of RET in the clinical landscape of breast cancer brain metastasis, especially within the estrogen receptor–positive patient subset, where its increased expression correlates with worse outcome.

### RET overexpression is a vulnerability in estrogen receptor–positive brain metastatic tumors

Having established a clinical role for RET in estrogen receptor–positive breast cancer brain metastasis and given that RET signaling typically activates proliferation-promoting pathways, we sought to determine whether pharmacological inhibition of RET affected the viability of brain metastatic cells. RET inhibitors with intercranial clinical activity LOXO-292 (selpercatinib) and BLU-667 (pralsetinib) statistically significantly reduced cell survival in RET^+^ breast cancer brain metastatic models (Supplementary Figure 2, A and B, available online). These differences between RET^+^ breast cancer brain metastatic models and controls were small, perhaps due in part to the absence of known RET fusions.

**Figure 2.**
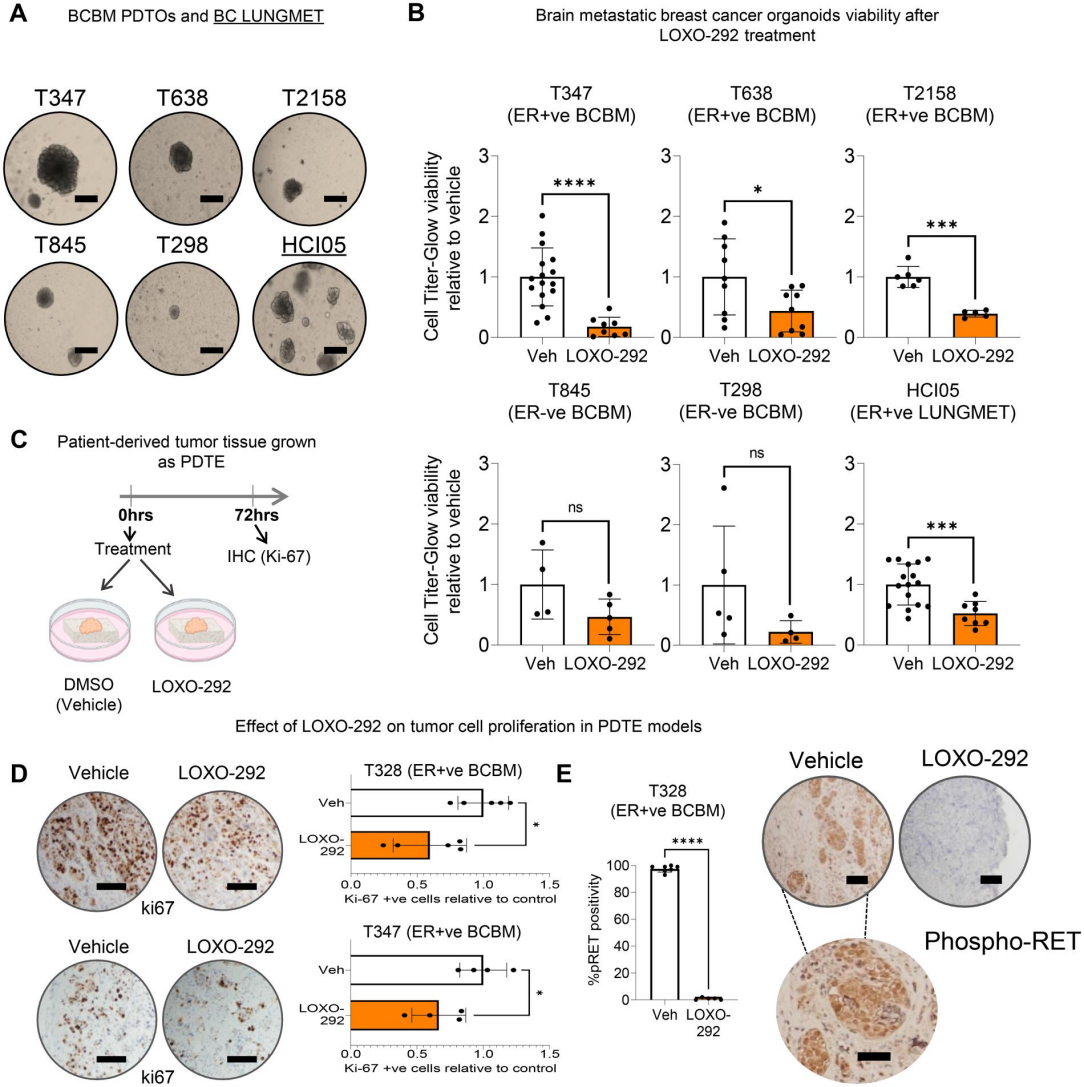
RET overexpression is a vulnerability in estrogen receptor–positive breast cancer brain metastatic tumors. **A**) Representative images of PDTOs. Individual organoids are shown (scale bar, 50 µm). **B**) Viability assessed by CellTiter-Glo kit showing luminescence (mean [SD]) after 7 days of Veh (DMSO) or LOXO-292 (10 µM) treatment in PDTOs. Two-tailed *t* test with Welch correction. *P* = ns, statistically non significant; *P <* *.05; ***P <* *.001; ****P <* *.0001 (n = 6 - 16, biological PDTO replicates). **C**) Graphical representation of establishment and treatment of PDTE. This scheme was created using elements from Biorender (https://biorender.com/). **D**) Ki-67% (proliferation index) analyzed by immunohistochemistry after treating PDTE with vehicle (DMSO) or LOXO-292 (10 µM) for 72 hours. Bar chart (mean [SD]) displays the percentage of Ki-67+ve cells (representative Ki-67 images shown on the left; scale bars, 100 μm). Two-sided unpaired *t* test with Welch correction. *P <* *.05 (n = 4, biological PDTE replicates). **E**) Relative phospho-RET protein expression; bar chart shows mean (SD) positivity after vehicle (DMSO) or LOXO-292 (10 µM) treatment for 72 hours, assessed by immunohistochemistry in a T328-PDTE. Two-sided unpaired *t* test with Welch correction. Scale bars, 100 μm. ****P <* *.001 (n = 6 - 8, biological PDTE replicates). BCBM = Breast Cancer Brain Metastasis; PDTOs = Patient Derived BCBM Organoids; PDTE = Patient Derived Tumor Explants; ER+ve = Estrogen-Receptor positive; ER-ve = Estrogen-Receptor negative; BC LUNGMET = lung metastasis of breast cancer; Ki-67+ve = Ki-67 positive cells; Veh = vehicle.

Efficacy of pharmacological inhibition of RET was tested in patient-derived tumor organoids (PDTO) (Figure 2, A; Supplementary Figure 2, C and D, available online). LOXO-292 induced statistically significant reductions in organoid viability in estrogen receptor–positive breast cancer brain metastasis models (T347-PDTO, T638-PDTO, and T2158-PDTO) and in an estrogen receptor–positive breast cancer RET+ve lung metastasis (HCI05-PDTO) (Figure 2, B). Though a response was observed in T298-PDTO and T845-PDTO estrogen receptor–negative breast cancer brain metastatic models, it was not statistically significant (Figure 2, B). We confirmed that the T347-PDTO, T328-PDTO, and HCI05-PDTO models, which are estrogen receptor positive and resistant to endocrine treatment, did not respond to tamoxifen and fulvestrant as expected (Supplementary Figure 2, E, available online). In patient-derived tumor explant breast cancer brain metastatic (PDTE) models (Figure 2, C), LOXO-292 treatment effectively reduced tumor viability in estrogen receptor–positive T328-PDTE and T347-PDTE, as determined by Ki-67 (Figure 2, D). Furthermore, we established that LOXO-292 inhibition of RET specifically targeted phospho-RET protein expression in T328-PDTE (Figure 2, E).

### Constitutively increased expression of RET mediates brain-specific metastasis development and homing

**Figure 3.**
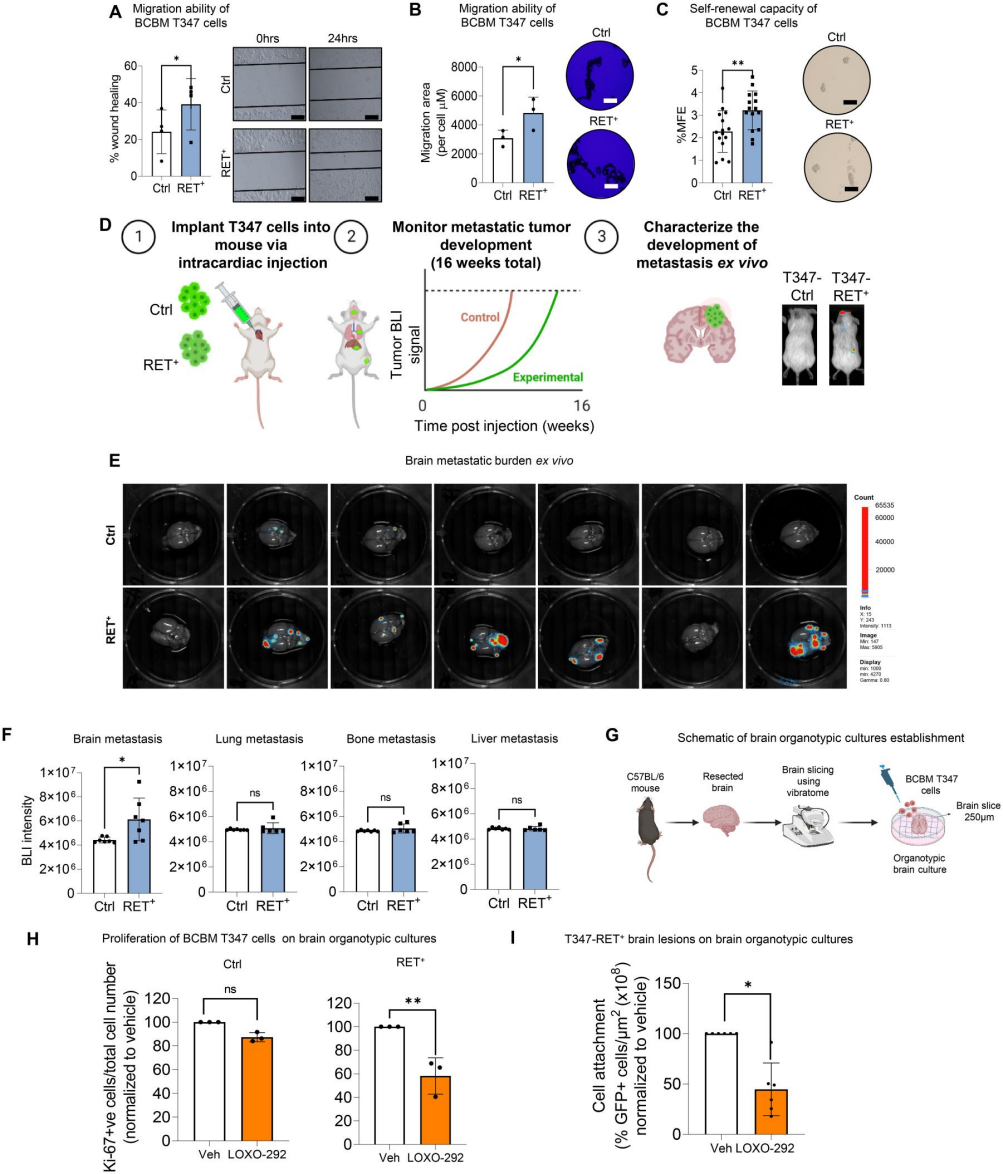
**Constitutively increased expression of RET mediates brain-specific metastasis development and homing. A**) *In vitro* migration ability of BCBM T347-Ctrl and T347-RET^+^ cells after 24 hours, measured with a wound healing assay (n = 4 biological replicates per cell line). Graph shows the percentage of wound closing compared to time zero (mean [SD]). Representative images (scale bar, 200 µm) at time 0 hours and 24 hours after the scratch was made. Two-sided paired *t* test. *P <* *.05. **B**) A Cellomics Cell Motility Kit was used to assess individual cell movement in collagen in a 96-well plate after 24 hours. Representative images of cells after fixing and staining (scale bars represent 200 μm). The mean migratory area per cell (μm) is shown on the graph (n = 3 biological replicates per cell line). Two-sided paired *t* test. *P <* *.05. **C**) Self-renewal capacity of T347-RET^+^ and T347-Ctrl cells was measured by mammosphere formation assay. Second-generation mammospheres (>50 µm) were counted under a microscope and presented as mammosphere formation efficiency (mean [SD]). Two-sided unpaired *t* test. **P <* *.005 (n = 3 biological replicates). **D**) Graphical representation of the experimental design. Mice were injected intracardialy with T347-Ctrl or T347-RET^+^ cells (n = 7 mice per cell line) and monitored over 16 weeks. This schematic was created using elements from Biorender (https://biorender.com/). **E**) At end of experiment (16 weeks) *ex vivo* brain BLI images were taken. **F**) Quantification of *ex vivo* brain BLI in brain, lung, bone and liver. The bar graph represents mean brain BLI values (n = 6 - 7 mice per cell line). Two-sided Mann-Whitney *t* test. *P* = ns, statistically non significant. *P <* *.05. **G**) Graphical representation of brain organotypic culture establishment. This scheme was created using elements from Biorender (https://biorender.com/). **H**) Ki-67% (proliferation index) analyzed by immunofluorescence after 72 hours of treatment of T347-Ctrl and T347-RET^+^ brain organotypic cultures with Veh (DMSO) or LOXO-292 (10 µM). Two-sided unpaired *t* test. *P* = ns, statistically non significant; **P <* *.01. (bar chart mean [SD]). **I**) Quantification of T347-RET^+^ cells’ attachment to the brain organotypic cultures after treatment with Veh (DMSO) or LOXO-292 (10 µM) (n = 6 brain organotypic cultures). BCBM cell lesion areas are normalized to the brain slice area for each replicate. Graph values are normalized to the control treated with DMSO. The bar chart shows mean [SD]. Two-sided Mann-Whitney *t* test, *P <* *.05. BCBM = Breast Cancer Brain Metastasis; Veh = vehicle; MFE = mammosphere formation efficiency; BLI = bioluminescence.

To further investigate the role of RET in breast cancer brain metastasis, we assessed the migratory ability of RET overexpressing breast cancer brain metastastatic cells (T347-RET^+^) (Supplementary Figure 3, A, available online). T347-RET^+^ cells displayed higher migration capacity than T347-Ctrl cells in both wound healing (Figure 3, A) and motility competence assay (Figure 3, B). Furthermore, T347-RET^+^ cells showed increased mammosphere formation ability compared with T347-Ctrl cells (Figure 3, C), which was inhibited with LOXO-292 (Supplementary Figure 3, B, available online).

We then determined whether RET overexpression is sufficient to enhance brain colonization. In a brain metastatic intracardiac mouse model, T347-RET^+^ cells displayed a greater capacity to colonize the brain than the T347-Ctrl cells (Figure 3, D and E). Although T347-RET^+^ cells were capable of colonizing other organs, RET was found to be essential for the specific growth of brain metastases (Figure 3, F). In murine organotypic brain cultures (Figure 3, G), which reproduce the characteristics and functions of brain metastatic cells *in vivo* (36), the addition of LOXO-292 resulted in decreased proliferation of T347-RET^+^ cells but not T347-Ctrl cells (Figure 3, H). Consistent with this finding, LOXO-292 also reduced T347-RET^+^ cell adherence to the brain (Figure 3, I; Supplementary Figure 3, C, available online). Taken together, these findings demonstrate that RET overexpression alone enhances brain metastatic competency of breast cancer cells, providing the first functional evidence of its role in this context.

### RET^+^ breast cancer brain metastatic cells display distinct pro–metastatic pathway activation

**Figure 4.**
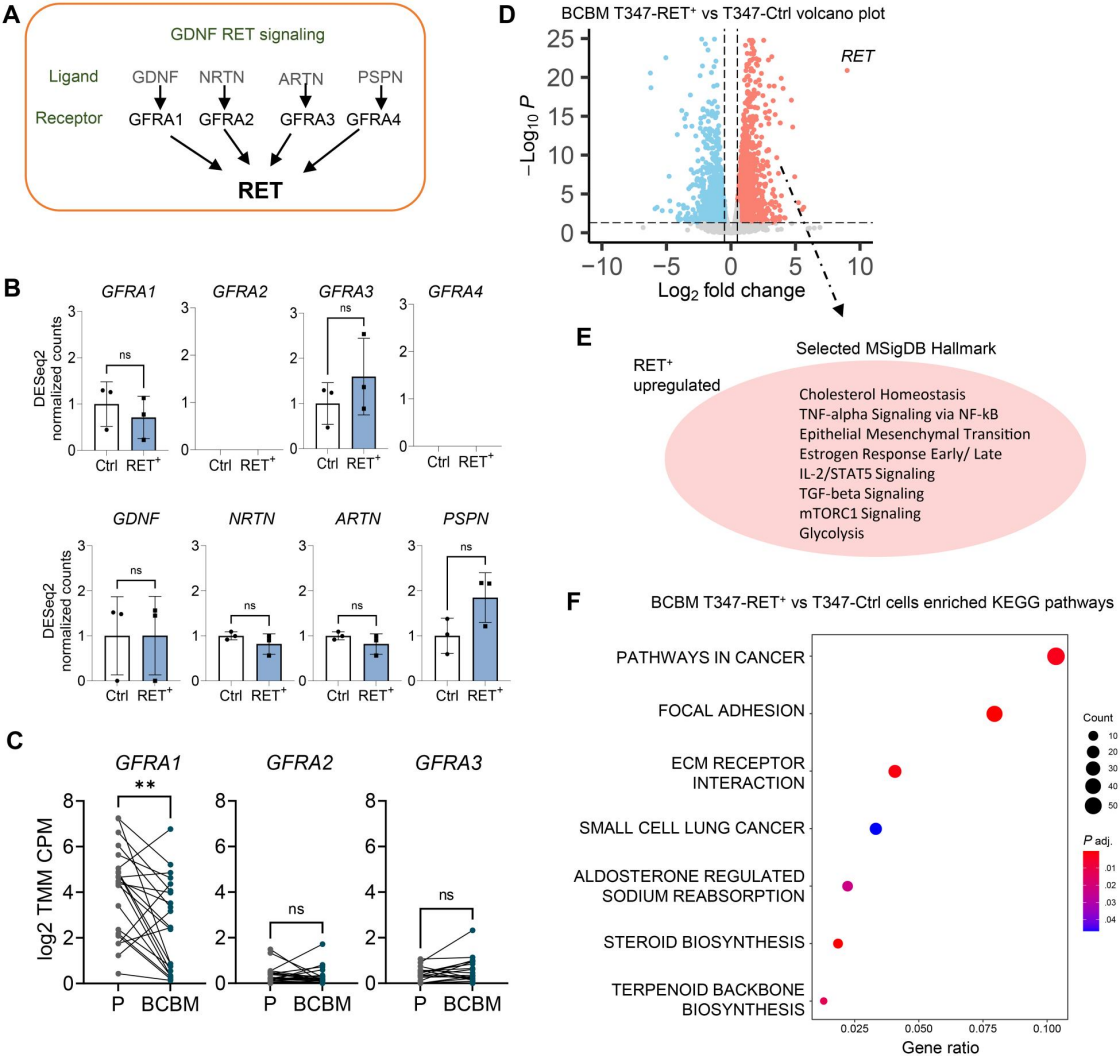
RET^+^ breast cancer brain metastatic cells display distinct pro–metastatic pathway activation (**A**) Graphical representation of the RET-GDNF signaling pathway. **B**) Gene expression of RET family receptors and soluble neurotrophic factor ligands in BCBM T347-Ctrl (Ctrl) and T347-RET^+^ (RET^+^) cells. **C**) Gene expression of GNDF family receptors in primary (P) breast cancer and BCBM (n = 22 patients). **D**) RNA sequencing was performed on T347-Ctrl and T347-RET^+^ cells. Differential gene expressions visualized with a volcano plot. **E**) Key MSigDb pathways found to be associated with a RET^+^ upregulated gene set are highlighted. **F**) KEGG pathway enrichment analysis was performed comparing RET^+^ with Ctrl gene expression (*P *<* *.05, log2 fold change > 0.5, pathway analysis of upregulated genes). GFRA 1-4 = GDNF Family Receptor Alpha 1-4; GDNF = Glial cell line-Derived Neurotrophic Factors; NTRN = Neurturin; ARTN = Artemin; PSPN = Persephin; P = Primary breast cancer; BCBM = Breast Cancer Brain Metastasis.

In terms of the RET mechanism of action, classical RET signaling involves GDNF family ligand binding to the RET/GFRA1 receptor (28). Surprisingly, in the brain metastatic setting, we found that *GFRA1* and key receptor-ligand partners in the known GDNF-RET signaling pathway were not consistently accompanied by the RET-driven transcriptome (Figure 4, A and B). *GFRA1-3* receptors were also not overexpressed in patients’ breast cancer brain metastatic samples, with *GFRA1* receptor expression higher in the primary tumor than in matched breast cancer brain metastatic samples (Figure 4, C). These data indicate that in this context, RET may employ alternative partners previously not reported in estrogen receptor–positive metastatic tumor cells.

To better understand the transcriptional signaling networks accompanied by RET overexpression, we undertook bulk RNA sequencing to compare the T347-Ctrl and T347-RET^+^ cells. Differential gene expression analysis revealed distinct RET expression–specific clustering of breast cancer brain metastastatic cells with statistically significant changes in the gene expression patterns, with 1866 genes elevated and 1568 genes repressed in the T347-RET^+^ cells (fold change > 0.5; *P*_adjusted_ < .05) (Figure 4, D; Supplementary Figure 4, A and B; Supplementary Table 2, available online). Functional annotation of differential genes expression in T347-RET^+^ cells by gene set enrichment analysis revealed genes upregulated in T347-RET^+^ cells associated with not only estrogen signaling but also with pro–metastatic pathways, including focal adhesion, epithelial mesenchymal transition, and TGF-beta signaling (Figure 4, E and F; Supplementary Figure 4, C and D; Supplementary Table 3 and 4, available online).

### RET overexpression in breast cancer mediates a brain-specific phenotype

**Figure 5.**
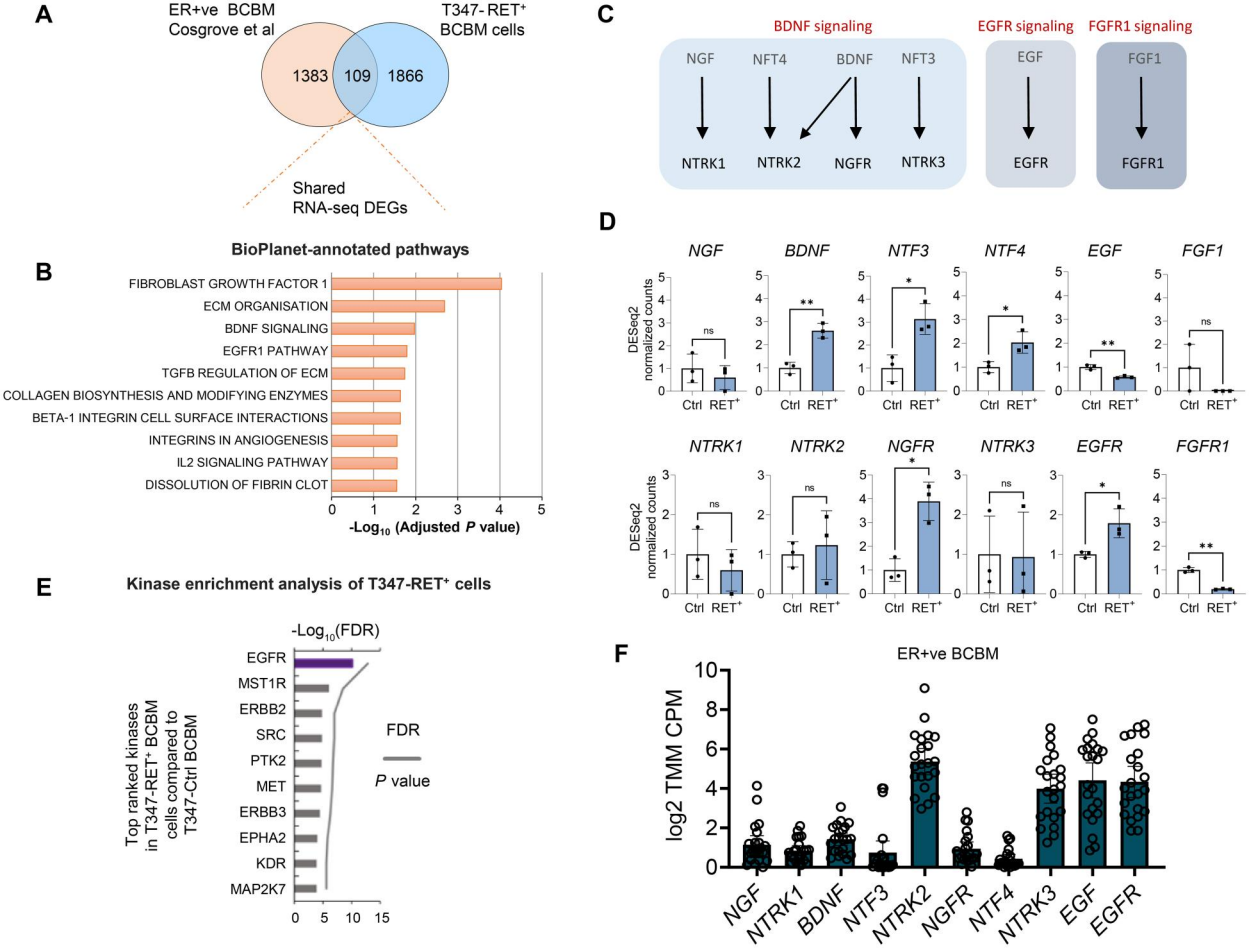
RET overexpression in breast cancer mediates brain-specific phenotype. **A**) Venn diagram showing 109 genes for the intersection between DEGs of ER+ve BCBM patient data (n = 22; log2 fold change > 2) and BCBM T347-RET^+^ cells (log2 fold change > 1) from RNA-seq data. **B**) BioPlanet-annotated pathways. The top 10 pathways based on adjusted *P* value are shown from overlapped genes (109 genes, from Figure 5, A). **C**) Graphical representation of BDNF and EGFR signaling neurothrophins and receptors. **D**) Gene expression of *BDNF*, *EGFR,* and *FGFR1* signaling receptors and ligands in T347-Ctrl and T347-RET^+^ cells. **E**) RNA-seq data from T347-RET^+^ versus T347-Ctrl cells was analyzed using Kinase Enrichment Analysis, version 3. The top 10 identified kinases are shown. **F**) Gene expression in ER+ve BCBM patients (n = 22). BDNF = brain-derived neurotrophic factor; DEGs = Differentially Expressed Genes; ER+ve = Estrogen Receptor–positive; BCBM = Breast Cancer Brain Metastasis; RNA-seq = RNA sequencing; EGFR = Epidermal Growth Factor Receptor; FDR = false discovery rate; NGF = Nerve Growth Factor; BDNF = Brain Derived Neurotrophic Factor; NTF3 = Neurotrophin 3; NTF4 = Neurotrophin 4; EGF = Epidermal Growth Factor; FGF1 = Fibroblast Growth Factor 1; NTRK1 = Neurotrophic Receptor Tyrosine Kinase 1; NTRK2 = Neurotrophic Receptor Tyrosine Kinase 2; NGFR = Nerve Growth Factor Receptor; NTRK3 = Neurotrophic Receptor Tyrosine Kinase 3; FGFR1 = Fibroblast Growth Factor Receptor 1.

As GDNF signaling did not appear to be a key RET partner in breast cancer brain metastases, we explored alternative associated receptor tyrosine kinases. Cross-referencing our RET upregulated gene set with the differential gene expression analysis from the clinical patient data in estrogen receptor–positive breast cancer brain metastasis (6), we identified 109 common genes (Figure 5, A; Supplementary Table 5, available online). The shared gene set was enriched for fibroblast growth factor 1 (FGF1), brain-derived neurotrophic factor (BDNF), and the EGF/EGFR signaling pathway (Figure 5, B; Supplementary Table 6, available online). EGF/EGFR’s role in tumorigenesis is well described (37). BDNF, a key neuronal development and survival factor (38), has also been previously linked with brain tumor progression and metastasis (39).

We investigated key receptor-ligands associated with these receptor tyrosine kinase pathways (Figure 5, C and D) and found BDNF-neurotrophic receptor tyrosine kinase 2 (NTRK2) and EGF-EGFR pairs to be most relevant based on enrichment in T347-RET^+^ cells. Using kinase enrichment analysis, we found EGFR to be the most enriched kinase in T347-RET^+^ breast cancer brain metastatic cells (Figure 5, E; Supplementary Table 7, available online). The significance of EGF-EGFR signaling in breast to brain metastasis progression was further substantiated by the ligand-receptor prominent expression in the breast cancer brain metastastatic tumor samples (Figure 5, F).

### RET^+^ breast cancer brain metastatic cells display distinct behavior and function

**Figure 6.**
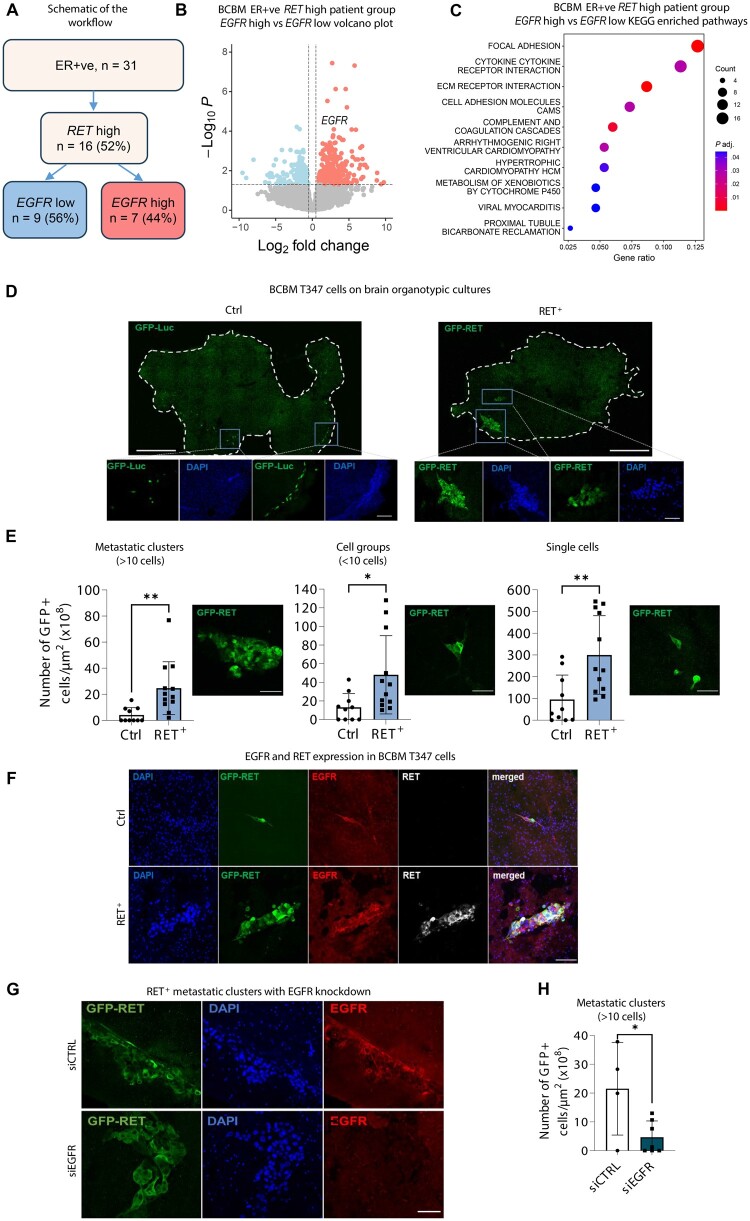
RET^+^ breast cancer brain metastatic cells display distinct behavior and function. **A**) The schematic workflow of ER+ve patients analysis based on their *RET* and *EGFR* gene expression in BCBM. The percentage of patients in the investigated population is shown in brackets. **B**) DEGs between the ER+ve BCBM *RET* high *EGFR* high (n = 7 patients) and *RET* high *EGFR* low (n = 9 patients) are visualized with a volcano plot. **C**) KEGG pathway enrichment analysis was performed comparing ER+ve BCBM *RET* high *EGFR* high to *RET* high *EGFR* low (*P *<* *.05, log2 fold change > 0.5, pathway analysis of upregulated genes). **D**) Representative immunofluorescence images of T347 metastatic clusters and cells (Ctrl and RET^+^) on brain organotypic cultures. DAPI - blue, T347-Ctrl-GFP-Luc or T347-RET^+^-GFP cells - green. The white line shows the borders of brain organotypic cultures. Whole-brain slice images are in the upper panel (scale bar, 1000 μm), and magnified images are in the lower panel (scale bar, 100 μm). **E**) Quantitative analysis of T347-Ctrl and T347-RET^+^ cell adherence. The number of formed metastatic clusters (>10 cells), number of groups (<10 cells) and single cells on the brain organotypic cultures (n = 3 biological replicates) were counted and presented per area of brain slice. Two-sided unpaired *t* test. *P <* *.05; **P <* *.01. Representative images of single cells, groups, and metastatic clusters are shown (scale bar, 100 μm). **F**) Representative immunofluorescence images showing RET and EGFR staining in T347-RET^+^ and T347-Ctrl cells in brain organotypic cultures (scale bar, 100 μm). **G**) Representative images of the immunofluorescence from Figure 6, H (scale bar 100 μm). **H**) Graph showing the number of formed T347-RET^+^ metastatic clusters at 72 hours on brain organotypic cultures (n = 3 biological replicates) after EGFR silencing. Two-sided unpaired *t* test. *P <* *.05. ER+ve = estrogen-receptor positive; EGFR = Epidermal Growth Factor Receptor; BCBM = Breast Cancer Brain Metastases; DEGs = Differential Expressed Genes; siEGFR = small interfering RNA for EGFR; siCTRL = small interfering RNA for control.

Given EGFR’s potential role in regulating cell mechanisms critical to breast cancer brain metastasis (5,12), we expanded our cohort to 31 estrogen receptor-positive breast cancer brain metastatic patients (Figure 6, A). Comparing gene expression between breast cancer brain metastasis *RET* high/*EGFR* high and *RET* high/*EGFR* low groups, we identified 429 upregulated and 252 downregulated genes (Figure 6, B). KEGG pathway analysis linked these upregulated genes to focal adhesion and ECM receptor interaction (Figure 6, C), suggesting a potential role for RET-EGFR in breast cancer brain metastatic adhesion, consistent with EGFR’s known cellular functions. We explored the EGFR–RET pair’s effect on cancer cell motility (Supplementary Figure 4, E, available online). T347-RET^+^ cells in the presence of BDNF, EGF, or growth factor–enriched serum showed increased invasion compared with controls (Supplementary Figure 4, F, available online), indicating the role of EGF-EGFR signaling in RET^+^ cell movement.

Although RET’s involvement in cell adhesion is known, its specific role in cancer cells adhering to the brain and breast cancer brain metastatic growth remains unexplored. Despite GDNF’s ability to regulate cellular adhesion in neuronal and glial cells (40), our data do not indicate a role for GDNF in RET-driven breast cancer brain metastasis. We investigated RET’s function in cancer cell brain adhesion using brain organotypic cultures with breast cancer brain metastatic cells (Figure 3, G). T347-RET^+^ cells showed statistically significant higher adhesion capacity than T347-Ctrl cells and demonstrated greater competency to form metastatic cell clusters (Figure 6, D and E). While single cells were attached to the brain surface, our data suggest that breast cancer brain metastatic cell clusters also integrated into the brain (Supplementary Figure 5, A, available online). Consistent with observations in our *in vivo* models, though cancer cell attachment was observed, enhanced cell adhesion and metastatic cluster formation in T347-RET^+^ cells were not evident in liver organotypic cultures, indicating brain-specific cluster formation (Supplementary Figure 5, B, available online).

**Figure 7.**
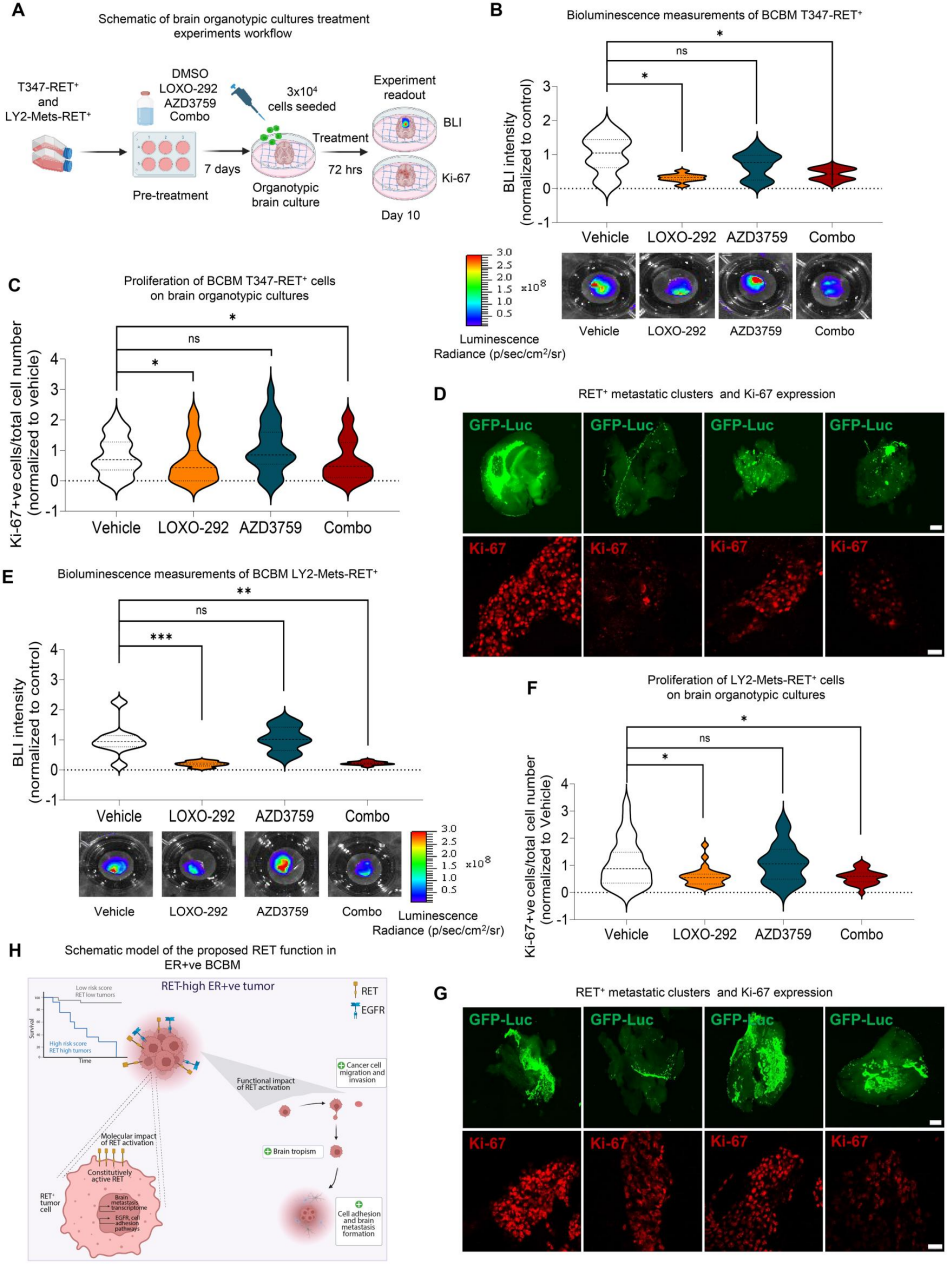
The effect of LOXO-292 and AZD3759 on breast cancer brain metastasis establishment. **A**) Schematic representation of experimental design. **B**) Upper panel: quantification of BLI signal (p/sec/cm^2^/sr) from T347-RET^+^ colonies on brain organotypic cultures after 10 days of treatment (7 days of pretreatment and 72 hours of treatment *ex vivo*). Violin plots show BLI signal distribution 72 hours after seeding cells on brain organotypic cultures normalized to vehicle (DMSO; n = 10 brain organotypic cultures per condition). Two-sided Mann-Whitney *t* test. *P* = ns, statistically non significant; *P <* *.05 (bar chart mean [SD]). Lower panel: representative images of BLI signal from T347-RET^+^ colonies treated with vehicle (DMSO), LOXO-292 (1 µM), AZD3759 (10 nM), or LOXO-292 and AZD3759 combination (Combo). **C**) Ki-67% (proliferation index) analyzed by immunofluorescence after 10 days of treatment of T347-RET^+ ^brain organotypic cultures. At least 2 images were counted per brain organotypic culture (n = 10 brain organotypic cultures per condition). Violin plots with median (dark dashed line) and quartiles (lighter dashed lines) show distribution of the graph values firstly normalized to total cell count per image and then normalized to vehicle control (DMSO). Two-sided Mann-Whitney *t* test. *P* = ns, statistically non significant; *P <* *.05 (bar chart mean [SD]). **D**) Representative immunofluorescence images from Figure 7, C. Upper panel: T347-RET^+^-GFP-Luc cell colonies (green) on brain organotypic culture (scale bar, 1000 μm). Lower panel: T347-RET^+^-GFP-Luc stained for Ki-67 (red; scale bar, 50 μm). **E**) Upper panel: quantification of BLI signal from LY2-Mets-RET^+^ colonies on brain organotypic cultures after 10 days of treatment (7 days of pretreatment and 72 hours of treatment *ex vivo*). Violin plots represent BLI after 72 hours normalized to vehicle (DMSO; n = 9 -12 brain organotypic cultures). Two-sided Mann-Whitney *t* test. *P* = ns, statistically non significant; **P <* *.01, ***P <* *.001 (bar chart mean [SD]). Lower panel: representative images of BLI signal from LY2-Mets-RET^+^ colonies on brain organotypic cultures after 10 days of treatment with vehicle (DMSO), LOXO-292 (10 µM), AZD3759 (10 nM), or LOXO-292 and AZD3759 combination (Combo). **F**) Ki-67% (proliferation index) analyzed by immunofluorescence after 10 days of treatment of LY2-Mets-RET^+^ brain organotypic cultures. At least 2 images were counted per brain organotypic culture (n = 10 brain organotypic cultures per condition). Values are normalized to the cell number per image. Final data are shown normalized to control vehicle (DMSO) as violin plots with median (dark dashed line) and quartiles (lighter dashed lines) of distribution displayed. Two-sided Mann-Whitney *t* test. *P* = ns, statistically non significant; *P <* *.05 (bar chart mean [SD]). **G**) Representative immunofluorescence images from Figure 7, F. Upper panel: LY2-RET^+^-GFP-Luc cell colonies (green) on brain organotypic cultures (scale bar, 1000 μm). Lower panel: Ki-67% representative images of LY2-Mets-RET^+^-GFP-Luc (red; scale bar, 50 μm). **H**) Schematic of RET function in ER+ve BCBM. BLI = bioluminescence; ER+ve = Estrogen-Receptor positive; BCBM = Breast Cancer Brain Metastasis.

Given EGFR’s crucial role in the RET-driven transcriptome here and its known collaborations with other receptors in cellular adhesion (41-43), we investigated its partnership with RET. The expression of EGFR was confirmed in both T347-Ctrl and T347-RET^+^ cells via immunofluorescence staining (Figure 6, F), and co-expression of RET and EGFR was observed in T347-RET^+^ metastatic clusters (Figure 6, F; Supplementary Figure 5, C, available online). Silencing EGFR in T347-RET^+^ cells decreased metastatic clusters without affecting single-cell adhesion (Figure 6, G and H; Supplementary Figure 5, D, available online). However the brain-penetrant EGFR inhibitor AZD3759 did not statistically significantly reduce T347-RET^+^ and endocrine-resistant/metastatic LY2-Mets-RET^+^ colonies both *in vitro* (Supplementary Figure 6, A-I, available online) and *ex vivo* (Figure 7, A-G). Moreover, LOXO-292 and AZD3759 combination drug treatment did not produce an additional effect (Supplementary Figure 6, D-I, available online), suggesting that AZD3759 did not further enhance the effect of LOXO-292 on breast cancer brain metastatic proliferation and colony formation in organotypic models (n > 9 brain slices) (Figure 7, A-G) nor in *in vitro* breast cancer brain metastatic cells (Supplementary Figure 6, D-I, available online). This disparity in effectiveness between AZD3759 and EGFR silencing may stem from AZD3759’s potentially lower efficacy in inhibiting EGFR wild-type signaling, especially in the context of RET and EGFR co-expression in breast cancer brain metastasis.

These data suggest that RET overexpression can cooperate with EGFR to create a pro–brain metastatic phenotype that facilitates the attachment of tumor cells to the brain (Figure 7, H).

## Discussion

Brain metastases are associated with poor prognosis and limited treatment options. Although the brain presents a suboptimal environment for tumor growth and colonization, certain breast tumors exhibit a greater propensity for metastasis to the brain. Previous studies have primarily focused on the triple-negative or HER2-positive models of breast cancer brain metastasis, resulting in an insufficient understanding of the mechanisms breast cancer brain metastasis in luminal (estrogen receptor–positive) tumors.

Here we identify RET as a putative mediator of breast cancer brain metastasis originating from estrogen receptor–positive breast cancer. Although the clinical associations between RET and survival in primary breast cancer have been inconsistent to date (14,44,45), we provide evidence that RET protein expression may serve as a biomarker for recurrence-free survival in estrogen receptor–positive primary tumors. Our analysis demonstrates the clinical relevance of RET, and its therapeutic potential is further supported by our experiments using patient-derived breast cancer brain metastatic explants and organoids.

Research has shown that when RET is activated, it can stimulate cell survival, movement, and the direction of nerve cell extensions by turning on specific signaling pathways (46). RET is also considered an estrogen-regulated gene (13,17,47,48), and we observed overrepresentation of estrogen signaling as well as focal adhesion and ECM receptor interaction pathways in RET^+^ cells, indicating the potential involvement of RET in breast cancer adhesion to the brain (49,50).

Although the role of RET as a driver of tumorigenicity and resistance to endocrine therapy is well established (16,44,45), our findings represent the first demonstration of its functional impact in facilitating the brain metastatic process, enhancing both cancer cell growth and formation of metastatic lesions. Of note, targeting RET with a selective inhibitor substantially reduced cancer cell brain metastatic potential, in particular, the formation of breast cancer brain metastastatic lesions.

RET signaling has widely been reported to be reliant on GFRA1 receptor/GDNF family ligand interactions (17,21,28,51). In this breast cancer brain metastases study, RET exploits a different signaling axis where the GFRA1 receptor and key GDNF-RET signaling partners were not consistently elevated in the RET overexpression group. This distinction may be important in the case of breast cancer brain metastases because blocking GDNF family ligand signals, essential for keeping dopaminergic neurons alive, could have consequential long-term effects on nerve health (52). Hence, it is crucial to balance the treatment of cancer while considering the potential consequences of blocking GDNF signaling.

Instead, our data suggest that RET^+^ breast cancer brain metastastatic cells exhibit BDNF and EGFR pathway upregulation. We uncover a functional relationship between RET and EGFR where both were found to contribute to cell adhesion of breast cancer cells to the brain in organotypic cultures. RET-EGFR interaction was previously described in a cell model of lung adenocarcinoma (53). Here, in brain metastatic clusters, we also observed the co-expression of EGFR and RET, but although genetic inhibition of EGFR was sufficient to limit the metastatic cell cluster formation on the brain, no pharmacological synergy was observed with combined RET and EGFR inhibition.

In *in vivo* patient-derived models, we demonstrated that RET overexpression alone was sufficient to enhance brain metastatic competency, presenting the first functional demonstration of its role in breast cancer brain metastasis. Given these findings, RET should be considered an attractive therapeutic target in a subset of breast cancer brain metastasis (not harboring *RET* mutations and fusion). Promising blood-brain barrier–penetrant targeted treatment options have emerged for patients with extracranial metastases that act on targetable EGFR and RET driver mutations. Additional clinical studies are needed to identify the patient subgroups that may benefit from RET inhibition with novel inhibitors such as BLU-667 and LOXO-292.

In conclusion, our research indicates that estrogen receptor–positive breast cancer cells can exploit RET activation to enhance their metastatic ability. Specifically, RET and EGFR aid the brain tropism of RET-expressing estrogen receptor–positive cells, promoting cell adhesion and metastatic cluster formation. The dynamic interaction between RET^+^ metastasizing breast cancer cells and co-activated signaling pathways is a critical factor in breast cancer brain metastasis formation. These results provide a strong scientific rational for the inclusion of RET inhibitors in the management strategy for breast cancer brain metastases.

## Supplementary Material

djae091_Supplementary_Data

## Data Availability

In compliance with institutional review board approvals and due to the lack of informed consent, patient-derived raw RNA sequencing data have not been publicly deposited. Raw RNA sequencing data for primary and metastatic sample pairs can, however, be provided upon request under regulatory compliance through a data usage agreement. Processed RNA sequencing data used for this study are available in the Gene Expression Omnibus under accession No. GSE184869 and GSE268217 (n = 16 breast cancer brain metastasis cases). Complete T347-Ctrl and T347-RET^+^ data generated for this study have been deposited in the Gene Expression Omnibus (GSE235886).
