# Supplementary material for: RET overexpression leads to increased brain metastatic competency in luminal breast cancer
Source: J Natl Cancer Inst. 2024 Jun 10;116(10):1632–44. doi: 10.1093/jnci/djae091 (PMC11461165; doi:10.1093/jnci/djae091)
Supplement: djae091_Supplementary_Data [file djae091_supplementary_data.zip › djae091_Supplementary_Data/FINAL-Jagust_et_al_Supplementary_Materials_JNCI_April.docx]

**Supplementary Material**

RET overexpression leads to increased brain metastatic competency in luminal breast cancer

Petra Jagust, PhD^1^ Aoibhin M. Powell, BSc^2^, Mihaela Ola, PhD^1^, Louise Watson, BSc^1^, Ana de Pablos-Aragoneses, MSc^3^, Pedro García- Gómez, PhD^3^, Ramón Fallon, MSc^1^, Fiona Bane, MSc^1^, Mona Heiland, PhD^3^, Gareth Morris, PhD^3,4^, Brenton Cavanagh, PhD^5^, Jason McGrath, PhD^1^, Daniela Ottaviani, PhD^1^, Aisling Hegarty, BSc^1^, Sinéad Cocchiglia, MSc^1^, Kieron J. Sweeney, MD^6^, Stephen MacNally, MD^6^, Francesca M. Brett, MD^6^, Jane Cryan, MD^7^, Alan Beausang, MD^7^, Patrick Morris, MD^8^, Manuel Valiente, PhD^3^, Arnold D.K. Hill, MD^1^, Damir Varešlija, PhD^2,8^* & Leonie S. Young, PhD ^1,8^*

**Corresponding Author**

Leonie S. Young, PhD., Department of Surgery, RCSI University of Medicine and Health Sciences, Dublin 2, Ireland, Tel: +353 1 4028515, [lyoung@rcsi.ie](mailto:lyoung@rcsi.ie)

* Joint senior authors: Leonie S Young, PhD., Department of Surgery, RCSI University of Medicine and Health Sciences, Dublin 2, Ireland, Tel: +353 1 4028576, [lyoung@rcsi.ie](mailto:lyoung@rcsi.ie) (corresponding author); Damir Varešlija, PhD., School of Pharmacy and Biomolecular Sciences, RCSI University of Medicine and Health Sciences, Dublin 2, Ireland, Tel: +353 1 4028659, [damirvareslija@rcsi.ie](mailto:damirvareslija@rcsi.ie).

**Supplementary Methods**

**Generation of breast cancer brain metastatic primary cells.** Breast cancer brain metastasis (BCBM) T347 Control (T347-Ctrl) cells were derived from a patient with an ER+ve, PR−ve, and HER2+ve breast cancer brain metastatic tumour as previously described (1). To establish GFP-Luc BCBM T347-Ctrl cells, cells were transduced (4x10^4^ cells in suspension + 8 µg/ml polybrene (Merck; #TR-1003)) with GFP-Luc lentivirus at a multiplicity of infection (MOI) of 5. BCBM T347-RET^+^ cells were derived by lentiviral transduction of BCBM T347-Ctrl cells with RET-GFP-Luc overexpression vector (Amsbio; #CLVP221-GP) or RET-RFP (Amsbio; #LVP221). 72 hrs post-transduction, cell media was replenished and enriched RET-GFP-Luc or RET-RFP positive cell population was selected by fluorescently activated cell sorting (FACS, BD FACSAria III) based on their GFP expression (Supplementary Figure 3, A). BCBM T347 cells were cultured in Cytiva Hyclone^TM^ DMEM/F12 (1:1) (Fisher Scientific; #SH3002301) media supplemented with 10 mM HEPES (Merck; #H3537), 5% FBS (Merck; #F6178), 0.5 μg/mL hydrocortisone (Merck; #H088), 1 mg/mL insulin (Invitrogen; #51500-056) and 1x Antibiotic-Antimycotic (Gibco; #11570486).

**Animal Studies**. To create patient-derived xenografts (used for PDTOs and PDTEs establishment), tumour tissue was fragmented in a sterile environment, and transplanted into the mammary fat pad of female NOD-SCID (NOD.CB17-Prkdc<scid>/NcrCrl); Charles River; RRID:IMSR_CRL:394) mice (*n* = 5). Estradiol was given as a supplement to ER+ve tumours. The HCI05 and HCI-011 models were provided as a gift from the Alana Welm lab. All patient-derived xenograft animal studies were approved by the Institutional Animal Care and Use Committee and the Health Products Regulatory Authority (HPRA). Metastatic animal studies protocols were approved by the animal welfare and ethical review committee, and all procedures were carried out under the guidelines of the Animal (Scientific Procedures) Act 1986.

For the intracardiac (IC) models of brain metastasis, 100 μl of phosphate-buffered saline (PBS, 1X) containing 1x10^5^ BCBM cells was injected into the left mouse ventricle (*n* =7 per condition). Prior to cancer cell detection by bioluminescence imaging (BLI), mice were anaesthetized using a mix of isoflurane and oxygen and injected with D-Luciferin (150 mg/kg) and imaged with Newton 7.0 machine (Vilber). The analysis of bioluminescence was conducted using Newton 7.0 imaging software.

**BCBM PDTE and PDX establishment**. All patient-derived xenograft (PDX) models have been previously described (2,3). Patient-derived tumour explants (PDTEs) established from BCBM PDX models were established as described previously (4).

**Immunohistochemistry.** Immunohistochemistry (IHC) staining of PDX and PDTE BCBM models was carried out in a laboratory. For IHC staining of BCBM-PDTEs, PDTEs were placed on gelatin dental sponges (Johnson&Johnson, #MS0005), pre-soaked in cell media, and treated with DMSO (vehicle; Merck; #D2650) or LOXO-292 (10 µM; gifted from LOXO-Oncology and Selleckchem, #S8782) for 72 hrs. PDTE BCBM models were then fixed in formalin, embedded in paraffin and sectioned for IHC staining. Primary antibody staining using Ready-to-use IHC (Biotin Free) Kit (Assay Genie; #BN00658) was carried out following sample deparaffinization and re-hydration. For performing heat antigen retrieval, antibody dependent, 1 mM EDTA solution in 10 mM Tris Base with 0.05% Tween 20 (pH 9) or 10 mM sodium citrate buffer (pH 6) were used. All primary antibodies were incubated for 1 hr at room temperature using recommended antibody dilutions, followed by corresponding biotin-labelled secondary antibody according to the manufacturer’s instructions. Patient-derived xenograft models (PDX) and patient-derived tumour explants (PDTE) BCBM models were stained with RET (Merck; #HPA008356;), phospho-RET (Y1062; Abcam; #ab51103; RRID:AB_870738) or Ki-67 (Clone MIB-1; Agilent; #M7240; RRID:AB_2142367) antibody.

Images were captured in cellSens Entry V1.8 using Olympus IX51 microscope equipped with a DP71 Camera. Immunohistochemistry slides were digitally imaged using Aperio ImageScope software (RRID:SCR_020993). Phospho-RET expression was quantified using Aperio positive pixel count algorithm, while nuclear protein expression was assessed by H-score. Total Ki-67 expression score was calculated as Ki-67 positively stained tumour cells nuclei per total number of cells in each image/field.

**Western blot analysis.** Whole-cell protein lysate was collected as previously described (2). Protein was quantified using a BCA kit (Thermo Fisher Scientific; #23227). Western blot was carried out using standard method. Proteins were separated by 10% SDS–PAGE, transferred to nitrocellulose membrane and blocked at room temperature in 5% bovine serum albumin (BSA; Merck; #A2153-100G) diluted in 0.1% Triton™-X 100 (Merck; #1.08603) TBS (Tris-Buffered Saline; TBS-T). Membranes were incubated overnight at 4°C with primary antibodies (mouse anti-human RET; Santa Cruz; #sc-365943; RRID:AB_10915753; β-actin (loading control); Merck; #A1978; RRID:AB_476692; Paxillin (D9G12) Rabbit mAb; Cell Signaling Technology; #12065; RRID:AB_2797814; SMAD3 antibody; Cell Signaling Technology; #9513; RRID:ab_2286450; anti-ITGA3; Sigma-Aldrich; #HPA008572; RRID:AB_2668094). Horseradish peroxidase (HRP)–tagged secondary antibodies were diluted in 5% BSA in TBS-T. Pierce enhanced chemiluminescence ECL substrate (Thermo Fisher Scientific; #32106) was used for protein detection.

**Organotypic brain and liver culture establishment**. Brains and livers from 2-6 months old female C57BL / 6 mice (Charles River; RRID:MGI:2159769) were used. In brief, organs were cut into 250 µm thick slices using vibratome (Campden Instruments LTD. 7000SMZ-2). Mouse brains were hemisected before sectioning. Slices were collected in Hanks’ balanced salt solution (Merck; #H9394) supplemented with 30 mM D(+)-glucose (Merck; #G8644), 2.5 mM HEPES (Merck; #H3537), 1 mM CaCl2 (Merck; #C-5080), 1 mM MgCl2 (Thermo Fisher Scientific; #AM9530G), 4 mM NaHCO3 (Merck; #C8532-500G) and washed 3 times in PBS with antibiotics (100 U/ml Penicillin-Streptomycin (Merck; #P4333) and 50 mg/ml Primocin; InvivoGen; #ant-pm-1) before placing them on 0.8 µm 19 mm membranes (Cytiva; #10417304) in BCBM T347 media. After 3 hrs, BCBM T347 cells (3x10^4^) were added on top of the organotypic slices in 2 µl of cell media and cultured at 37°C for up to 3 days.

**LOXO-292 and AZD3759 treatment ex vivo (organotypic brain culture).** For intervention experiments, DMSO (vehicle) or LOXO-292 (10 µM) was added to the brain organotypic cultures at day zero and the media was changed every day. Transfection with siEGFR was repeated before adding siEGFR T347-RET^+^ cells to brain organotypic cultures. After 72 hrs organotypic cultures were fixed overnight at 4°C in 4% paraformaldehyde (PFA; pH 7; Merck; #158127). Slices were then washed 3 times in PBS and free-floating immunofluorescence was performed.

For combination treatment study (**Figure 7, A**) T347-RET^+^ and LY2-Mets-RET^+^ cells were pre-treated with DMSO, LOXO-292 (1 µM for T347-RET^+^ and 10 µM for LY2-Mets-RET^+^ cells), AZD3759 (MedChemExpress; #HY-18750) (10 nM for T347-RET^+^ and 10 nM for LY2-Mets-RET^+^), or LOXO-292 and AZD3759 combination for 7 days. After obtaining brain slices (following protocol above, see Organotypic brain and liver culture establishment), 3x10^4^ cancer cells were placed on the surface of the brain slices and DMSO, LOXO-292, AZD3759 or combination treatment was continued for an additional 72 hrs. Bioluminescence (BLI) was measured at day 10, 72 hrs after seeding on the brain slices, using IVIS Lumina III imaging system (PerkinElmer). Brain organotypic cultures were then fixed in 4% PFA overnight at 4°C and free-floating 3D immunofluorescence staining was performed following the protocol below (see Free-floating 3D immunofluorescence).

**Free-floating 3D immunofluorescence****.** Organotypic cultures obtained after 4% PFA fixation were blocked at room temperature for 2 hrs with 10% normal goat serum (Abcam; #ab7481), 2% BSA (Merck; #A9418) and 0.25% Triton™ X-100 (Merck; #1.08603) in PBS with agitation following a published protocol (5). Slices were stained RET (c-3, Santa Cruz, sc-365943; RRID:AB_10915753), GFP (Aves Labs, GFP-1020), EGFR (D3B1; Cell Signaling Technology; #4267; RRID:AB_2246311), DAPI (Merck; #D9542) bisBenzimide H 33258 (Merck; #B2883 and Ki-67 (Agilent; #M7240; RRID:AB_2142367 or Abcam; #ab15580; RRID:AB_443209) antibodies diluted in blocking buffer overnight at 4°C and 30 min at room temperature the next day with agitation. Brain and liver slices were then washed 6 times (5 min each wash with agitation) with 0.25% Triton-PBS and incubated with the corresponding fluorescent secondary antibody (Thermo Fisher Scientific; Goat anti-Mouse IgG (H+L) Highly Cross-Adsorbed Secondary Antibody, Alexa Fluor™ 647; #A-212360; RRID:AB_2535805; Goat anti-Rabbit IgG (H+L) Cross-Adsorbed Secondary Antibody, Alexa Fluor™ 594; #A-11012; RRID:AB_2534079; Goat anti-Chicken IgY (H+L) Secondary Antibody, Alexa Fluor™ 488, #A-11039; AB_2534096) in blocking solution for 2 hrs at room temperature, followed by 4 washes in 0.25% Triton-PBS wash. Finally, DAPI (Merck; #D9542) was used for nuclear staining. After 30 min incubation with DAPI at room temperature, organotypic cultures were washed 2 times with 0.25% Triton-PBS and mounted on microscope slides using DAPI mounting media (Thermo Fisher Scientific; #P36931).

**Microscopy image acquisition and analysis.** Zeiss LSM710 confocal, Celldiscoverer 7 microscope and an Olympus IX51 widefield microscope were used to acquire images. For image acquisition the confocal microscope was equipped with a Plan-Neofluar 2.5x/0.075, W N-Achroplan 10x/0.3 and W N-Plan-Apochromat 20x/1.0 objectives. The UV fluorescence was excited using 405 nm (detection range 410 – 516 nm), green fluorescence was excited using 488 nm (detection range 494 – 572 nm), red fluorescence was excited using 561 nm (detection range 572 - 640 nm) and far-red fluorescence was excited using 633 nm (detection range 638 - 755 nm) lasers. The Olympus was equipped with Zeiss Plan-Apochromat 5x/0.16, and Olympus UplanFL N 10x/0.3 and 20x/0.5 objectives and standard DAPI, FITC and TRITC bandpass filters. A Celldiscoverer 7 microscope equipped with a 5×/0.35 numerical aperture Plan-Apochromat objective and 0.5x optovar was used to record full brain slice fluorescent images with an Axiocam 506 camera. All tile images were stitched and fused in Zen blue ed. software ver. 3.1. All immunofluorescence images were analysed and prepared with ImageJ (RRID:SCR_003070) or Fiji software (6) (RRID:SCR_002285). The percentage of cancer cells positive for Ki-67 after 72 hrs of LOXO-292 (1 µM or 10 µM), AZD3759 (10 nM and 100 nM) and LOXO-292 and AZD3759 combination treatments in brain organotypic cultures was calculated as a percentage of total number of cancer cells (based on DAPI staining) in each field. At least two fields per treatment were used for scoring.

**RNA interference.** Inhibitor RNA (siRNA; On-target plus SMART pool) siEGFR (#L-003114-00-0005) and non-targeting (siCTRL, #D-001810-10-20) were obtained from Dharmacon™. 24 hrs prior to transfection, BCBM T347-RET^+^ cells were seeded into 6-well plates in BCBM T347 cell media. At 70-80% confluence, the cells were incubated in antibiotic-free media. Cells were then transfected with 30 nM siRNA using Lipofectamine 2000 transfection reagent (Invitrogen; #11668019) according to the manufacturer's instructions. After 72 hrs cells were harvested by 1X trypsin-EDTA (Merck; #F4174) and subsequent experiments were performed.

**Mammosphere formation assay.** BCBM mammospheres were established as previously described (7). 5x10^3^ BCBM T347-Ctrl and T347-RET^+^ cells were seeded in a low attachment 6-well plate (Corning; #3471) in DMEM F12- phenol red free (Gibco; #21041-025) media with 2% B27 supplement (Gibco; #14504044), 20 ng/ml hFGF (Peprotech, Cat number 100-18B), 20 ng/ml hEGF (Peprotech; #AF 100-15), 5 µg/ml heparin (Alfa Aesar; #A16198) and 100 U/ml Penicillin-Streptomycin (Merck; #P4333). After 7 days, formed mammospheres were disintegrated and re-seeded at 2.5x10^2^ cells/ml density to form second-generation mammospheres. To assess the self-renewal capacity of BCBM T347 cells, formed second-generation mammospheres (> 50 µm) were counted after 5 days using a microscope (Olympus IX51 widefield microscope). Mammosphere formation ability was calculated as the number of formed spheres (after 5 days) in the second generation/number of seeded cells *100.

**Inhibition of mammosphere formation assay.** Cells were cultured under 3D forming conditions as described in the previous paragraph. LOXO-292 (10 µM) or DMSO (vehicle) were added to the cell media when second-generation mammospheres were seeded. Cells were grown for additional 5 days before assessing their self-renewal capacity as described in the previous paragraph.

**Transwell invasion assay**. Transwell invasion assay was performed using Boyden chamber assay (Corning, #354480) as per manufacturer instructions. BCBM T347 cells were seeded on top of the matrigel-coated inserted (upper chamber) in serum-free media and left to migrate through the membrane for 24 hrs towards the lower chamber containing media supplemented with 10% Fetal Bovine Serum (FBS), BCBM T347 media with the addition of 100 µg/ml brain-derived neurotrophic factor (BDNF, Peprotech, #450-02) or 100 ng/ml EGF (Peprotech, #AF 100-15). Invading cells were counted under the microscope (Olympus IX51 widefield microscope).

**Clonogenic assay**. Cells were seeded in 6-well plates (Greiner; #07-000-208) at density 1.5x10^3^- 4x10^3^ per well. Cells were treated with LOXO-292 (T347-Ctrl and T347-RET^+^; 100 nM, 1 µM, 5 µM, 10 µM, 20 µM. LY2-Mets and LY2-Mets-RET^+^; 1 nM, 10 nM, 100 nM, 1 µM, 10 µM), AZD3759 (T347-RET^+^ and LY2-Mets-RET^+^; 10 nM, 100 nM, 500 nM, 1 µM, 5 µM) and BLU-667 (Selleckchem; #S8716; T347-RET^+^; 1nM, 100nM, 1 µM, 10 µM, 20 µM) on Day 1 and treatments were replenished every 72 hrs. After 10-14 days of incubation, the cells were fixed and stained in 0.5% crystal violet solution (60% w/v methanol, 40% v/v deionised water). The colonies were counted using GelCount instrument (Oxford Optronix, Ltd., Oxford, UK) and optimised via the compact Hough and radial map (CHARM) image processing algorithms for each cell line. The plating efficiencies (PE) were calculated using the formula: PE = number of colonies counted/number of cells seeded. The surviving fraction (SF) was calculated using the formula: SF = number of colonies counted/PE × number of cells seeded.

**Clonogenic assay for drug synergy**. For calculation synergy cells were seeded in 12-well plates (Sarstedt; #83.3921.005) at density 1.2x10^3^- 2.2x10^3^ per well and treated with LOXO-292 and AZD3759 for 7-10 days before being fixed and stained with crystal violet solution (details above). ZIP and Bliss synergy scores were calculated using the SynergyFinder+ web application (8), while the Bliss score surface graph was produced with function PlotSynergy (type 3D, method Bliss, custom colours) in R package synergyfinder (v3.6.3).

**Cell motility assay.** Cell motility assay was performed as previously described (9). 96-well plate was pre-coated with collagen type I (Corning; #354236) as per manufacturer instructions of the Cellomics Cell Motility Kit (Thermo Fisher Scientific; #K08-0001-1). BCBM T347-Ctrl and T347-RET^+^ cells were seeded at 250 cells/well density and their ability to migrate through collagen was measured after 24 hrs using ImageJ software (RRID:SCR_003070).

**Wound healing assay.** BCBM cells were grown to >80% confluence, forming a monolayer in a 24-well plate. A linear wound was created with a sterile pipette tip (20-200 µM tip) in the centre of the wells at day zero. Afterwards, wells were washed in PBS (2x) and BCBM T347 cell media was added. Light phase microscopy (Olympus IX51 widefield microscope) was used to monitor wound closure (5x magnification). Images of the cells migrating into the wound were taken at time zero and 24 hours after. Up to 4 fields per well were imaged to cover the whole wound. ImageJ software (RRID:SCR_003070) was used for analysis and measurements of the wounds closing. Wound closing percentage was calculated as (initial wound area - final wound area)/initial wound area) x 100.

**Extraction protocol and RNA-sequencing.** RNA was extracted from formalin-fixed paraffin-embedded (FFPE) tissue obtained from patient-matched primary breast tumours and resected brain metastases, employing the Qiagen AllPrep DNA/RNA FFPE kit (Qiagen, #80234) on a QIAcube instrument following standard protocols. Subsequently, sample quality and concentration were evaluated using 2100 Expert Software - Bioanalyzer System. Library preparation was performed using 100 ng of total RNA and NUSeq Core Facility of Northwestern University's Stranded Illumina Total RNA-seq library preparation protocol (NUSeq, #NGS-3). Indexed, pooled libraries were then sequenced on an Illumina NovaSeq 6000 (paired-end reads, 2 x 150 bp). Information regarding the acquisition of samples, tissue processing, and RNA-sequencing library preparation for patient-matched primary breast and brain metastatic tumor samples from n = 45/63 patients (n = 90 samples; PITT-RCSI-MAYO Cohort) is provided in Cosgrove et al. (2).

BCBM T347-Ctrl and T347-RET^+^ cells (*n* =3) were collected, RNA was extracted using Qiagen RNeasy Kit (Qiagen; #74104) and subjected to 100bp PE sequencing using the BGISEQ 500 platform. BGI Genomics (Hong Kong) carried out initial data pre-processing using BGI internal software SOAPnuke (v2.2.5,10) to remove adaptors, reads containing more than 0.1% unknown bases (N) and low-quality reads. Quality control metrics to remove adapter sequences and low-quality reads of the paired-end clean sequencing reads was performed and assessed by using FASTQCFastQC.

**Differential gene expression analysis**. Salmon workflow with seqBias and gcBias corrections was used for quasi-mapping and quantification of RNA-seq transcript abundance in both cell line and patient derived RNAseq and using a 31bp k-mer index of the GRCH38 genome build (11). DESeq2 package (12) was used to calculate differential gene expression between T347-RET^+^ and T347-Ctrl control, respectively between ER+ve RET high EGFR high expressing patients and RET high EGFR low expressing patients, using log2FC=0.5 and FDR=0.05 as cut-off for significant resultss. EnhancedVolcano R package (v1.16.0) [https://github.com/kevinblighe/EnhancedVolcano] was used to plot differentially expressed genes (and to highlight key regulators and their position within the differentially expressed genes). The enrichGOr function from ClusterProfiler R package (v4.6.0) with minGSSize = 10, maxGSSize = 500, ont = “BP” and FDR = 0.05 was used in conjunction with the msigdbr package (v7.5.1) (species = "Homo sapiens", category = "C2", subcategory = "CP:KEGG") to functionally annotate differentially expressed genes with a log2FC > 0.5 and adjusted p-value < 0.05, according to Kyoto Encyclopedia of Genes and Genomes (KEGG) GO biological process termspathways. Functional enrichments results were plotted using the dotplot function from Enrichplot R package (v1.18.1).

**OncoPrint visualisation of clinically actionable kinases.** The gene expression counts for the paired primary and metastatic samples of 22 patients with ER+ve were calculated from sequencing. Gene expression differences were calculated using the edgeR package, where counts are normalized to effective library size and then normalized to CPM (counts per million) after which the log2 is taken. The log2 of the difference between primary and metastatic values is then taken to give log fold changes in gene expression. An expression gain was then defined as an increased expression state occurring in the 95th percentile of all expression gains (log2FC = 1.934) so that patients that underwent strong expression gains for certain genes could then be highlighted. The Drug Gene Interaction Database (DGIdB 2.0) was used to identify clinically actionable kinases, and these were extracted from the expression gain matrix, except genes NTRK2 and NTRK3 which have been shown to have frequent high expression in primaries. The OncoPrint function from the ComplexHeatmap package was then used to graph recurrent (n > 1) gains on a patient-by-patient basis in order of the genes with most gains in patients. Percentages reflect how many of the total patients have gains in each clinically actionable kinase. A bar chart on the right of the OncoPrint gives the total number of these gains per kinase.

**Real-Time Quantitative Polymerase Chain Reaction (RTqPCR).** RNA was extracted using using Qiagen RNeasy Kit (Qiagen, #74104) according to the manufacturer’s instructions. RNA concentration and purity were determined by spectrophotometry (Nanodrop-800; Thermo Fisher Scientific). RET expression was assessed by TaqMan RT-qPCR assay (Hs01120030_m1; Applied Biosystems; StepOne real-Time PCR System; Thermo Fisher Scientific).

**Data analysis.** Kaplan-Meier estimates of breast cancer-specific overall survival (OS) were determined for high RET expression and low RET expression (*n* = 820). The OS time for each patient was calculated in years from the date of diagnosis (when the diagnosis date was not available, the primary surgery date was used) until the date of death or censoring. Kaplan-Meier estimates were determined using the survfit function in the survival R package (v3.4-0) and visualized using the ggsurvplot function in the survminer R package (v0.4.9). The upper quartile value (300) was selected as cut-off point to define high and low RET expression levels.

**Supplementary References**

1. Browne AL, Charmsaz S, Varešlija D, et al. Network analysis of SRC-1 reveals a novel transcription factor hub which regulates endocrine resistant breast cancer. *Oncogene*. 2018;37(15):2008-2021.

2. Varešlija D, Ward E, Purcell SP, et al. Comparative analysis of the AIB1 interactome in breast cancer reveals MTA2 as a repressive partner which silences E-Cadherin to promote EMT and associates with a pro-metastatic phenotype. *Oncogene 2021 407*. 2021;40(7):1318-1331.

3. Cosgrove N, Varešlija D, Keelan S, et al. Mapping molecular subtype specific alterations in breast cancer brain metastases identifies clinically relevant vulnerabilities. *Nat Commun 2022 131*. 2022;13(1):1-16.

4. Varešlija D, Priedigkeit N, Fagan A, et al. Transcriptome characterization of matched primary breast and brain metastatic tumors to detect novel actionable targets. *J Natl Cancer Inst*. 2019;111(4):388-398.

5. Zhu L, Valiente M. Organotypic brain cultures for metastasis research. *Neuromethods*. 2021;158:119-132.

6. Schindelin J, Arganda-Carreras I, Frise E, et al. Fiji: an open-source platform for biological-image analysis. *Nat Methods 2012 97*. 2012;9(7):676-682.

7. Charmsaz S, Doherty B, Cocchiglia S, et al. ADAM22/LGI1 complex as a new actionable target for breast cancer brain metastasis. *BMC Med*. 2020;18(1):349.

8. Zheng S, Wang W, Aldahdooh J, et al. SynergyFinder Plus: Toward Better Interpretation and Annotation of Drug Combination Screening Datasets. *Genomics, Proteomics Bioinforma*. 2022;20(3):587-596.

9. Kang R, Zeh HJ, Lotze MT, Tang D. The Beclin 1 network regulates autophagy and apoptosis. *Cell Death Differ*. 2011;18(4):571-580.

10. Chen Y, Chen Y, Shi C, et al. SOAPnuke: a MapReduce acceleration-supported software for integrated quality control and preprocessing of high-throughput sequencing data. *Gigascience*. 2018;7(1):1-6.

11. Patro R, Duggal G, Love MI, Irizarry RA, Kingsford C. Salmon: fast and bias-aware quantification of transcript expression using dual-phase inference. *Nat Methods*. 2017;14(4):417-419.

12. Love MI, Huber W, Anders S. Moderated estimation of fold change and dispersion for RNA-seq data with DESeq2. *Genome Biol*. 2014;15(12):550.

**Supplementary Tables**

**Supplementary Table 1:** Association analysis between ER and HER2 patient status, and RET IHC score (TMA data)

| **Clinical parameter** | ***n* (total)** | **RET high (=300)** | **RET low (<300)** | ***P* value**  **Fisher exact** |
| --- | --- | --- | --- | --- |
| ER+ve | 661 | 386 | 275 | <0.0001 |
| ER-ve | 159 | 65 | 94 |  |
| HER2+ve | 131 | 88 | 43 | 0.002 |
| HER2-ve | 684 | 359 | 325 |  |

**Supplementary Figures**

**
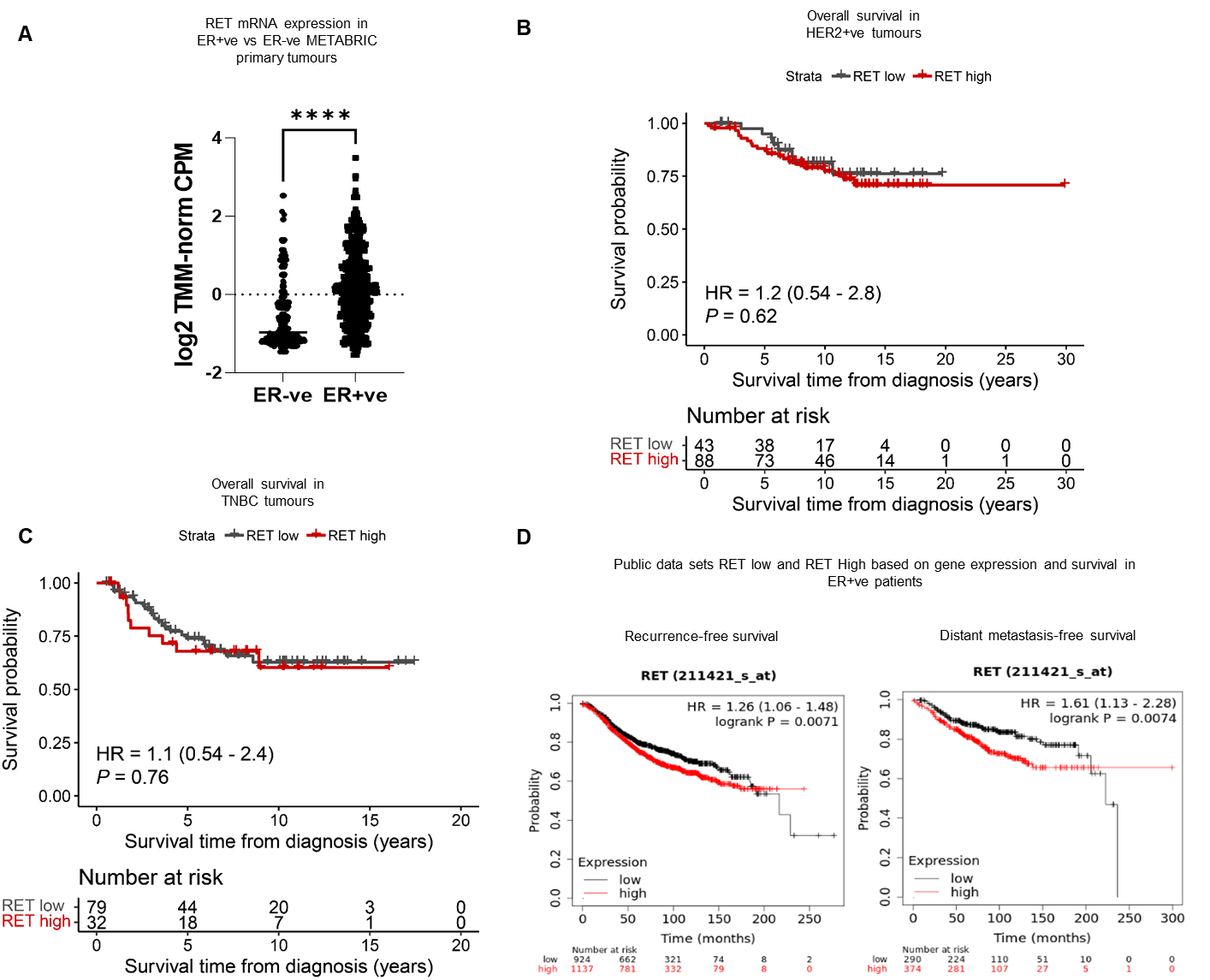
**

**Supplementary Figure 1**. **RET expression in ER+ve primary breast cancers and correlation with survival in publicly available datasets.** **A**. *RET* mRNA expression in ER+ve and ER-ve primary breast cancer tumours from the METABRIC cohort (two-tailed t- test, *P* < 0.0001; *n* = 1028). **B-C**. Kaplan–Meier analysis of overall survival in (**B**) HER2+ve (*n* = 131) and (**C**) triple negative breast cancer (TNBC) tissue (*n* = 111). IHC cut-off score of 300 for RET was obtained with ROC curve. **D**. *RET* mRNA expression was correlated with recurrence and distant metastasis-free survival of breast cancer patients using the KM-Plotter. *RET* expression was stratified as High vs. Low according to an auto-select best cut-off value, and survival plots based on previously published data sets were generated using http://kmplot.com. *P* values were calculated by the Logrank test.


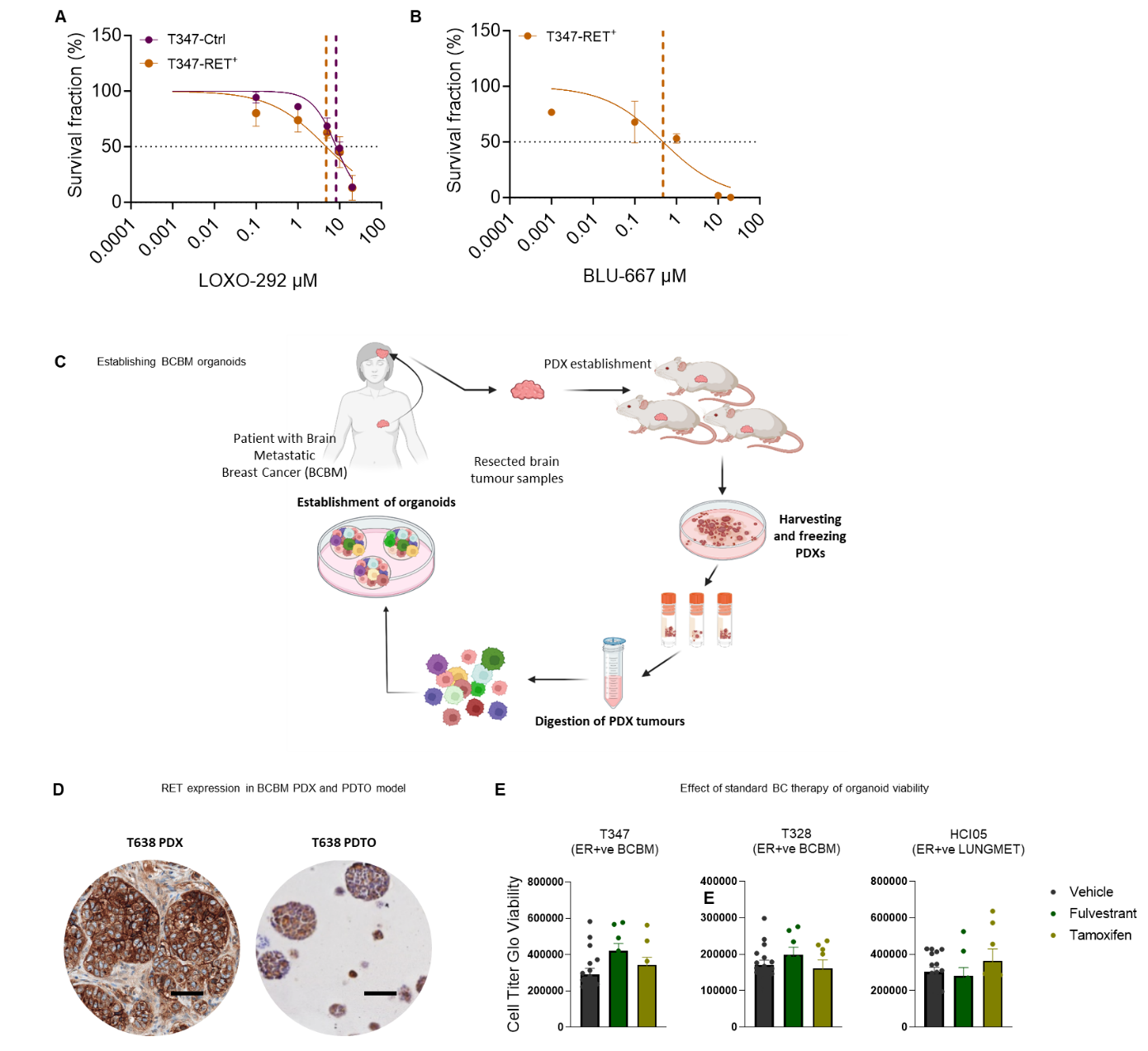


**Supplementary Figure 2. Generation and characterization of PDX-derived BCBM tumour organoids. A**. Graphical representation of LOXO-292 dose-response curves in T347-Ctrl and T347-RET^+^ cells measured by clonogenic assay. A dotted vertical line shows half maximal inhibitory concentration (IC50) for each cell line (*n* = 3 biological replicates). The T347-Ctrl cell line has an IC50 value of 8.253 μM and a T347-RET^+^ cell line of 4.782 μM. Data is presented as mean ± S.D. **B**. Graphical representation of BLU-667 dose-response curves in T347-RET^+^ BCBM cell line. A dotted vertical line shows half maximal inhibitory concentration (IC50) (*n* = 3 biological replicates). T347-RET^+^cell line has an IC50 value of 0.4775 μM. Data is presented as mean ± S.D. **C.** Graphical representation of PDX-derived BCBM organoids (PDTOs) models. This schematic was created using elements from Biorender (<https://biorender.com/>). **D**. Representative immunohistochemistry (IHC) images of patient-derived BCBM xenografts (PDXs) T638 model (scale bars, 100 μm) and PDTO-T638 model (scale bar 50 µm) stained for RET protein. **E**. Viability assessed by CellTiter-Glo showing luminescence (mean ± S.D.) after 7 days of vehicle (Veh, DMSO) or Fulvestrant (100 nM) and Tamoxifen (0.1 µM) treatment in PDTOs. Two-tailed t-test with Welch’s correction. *P* = ns, non-significant


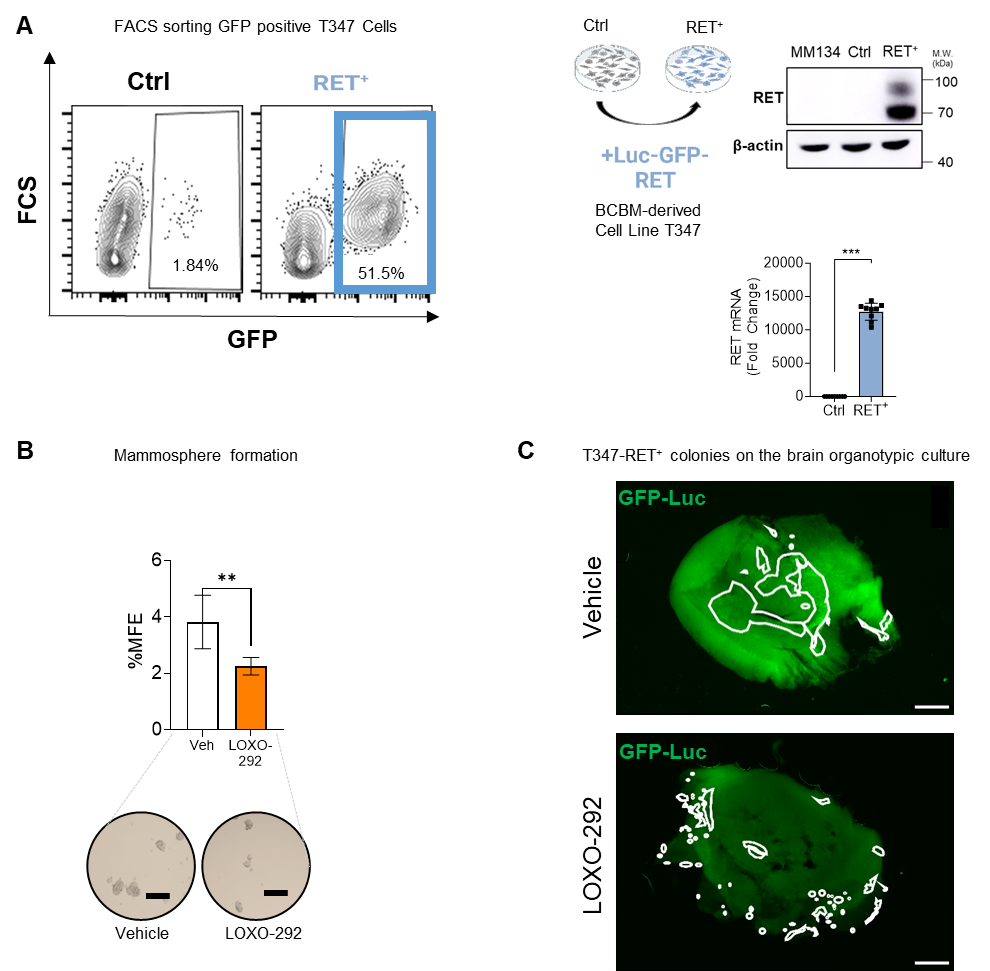


**Supplementary Figure 3. Establishment and characterization of BCBM T347-RET^+^ cell line. A.** BCBM T347 cells were transduced with RET-GFP-Luc lentivirus and fluorescence-activated cell sorted (FACS) based on GFP expression. Depicted is mRNA gene expression in T347-RET^+^ compared to T347-Ctrl cells. Western blot of RET protein expression, comparing RET expression in T347-RET^+^ versus T347-Ctrl and ER+ve cell line, MDA-MB-134-VI (MM134). **B**. Bar graph (means ± S.D.) showing MFE following vehicle (Veh, DMSO) or LOXO-292 (1 µM) treatment for 5 days in T347-Ctrl or T347-RET^+^ mammospheres (*n* ≥ 3). Two-tailed unpaired t-test. **, *P* < 0.005; (*n* = 3 biological replicates; scale bar 200 µm). **C**. Representative images of T347-RET^+^ colonies on brain organotypic cultures after 72 hrs of treatment with vehicle (DMSO) or LOXO-292 (10 µM). White lines mark BCBM cell colonies. Related to Figure 3, I. Scale bar 1000 μm.


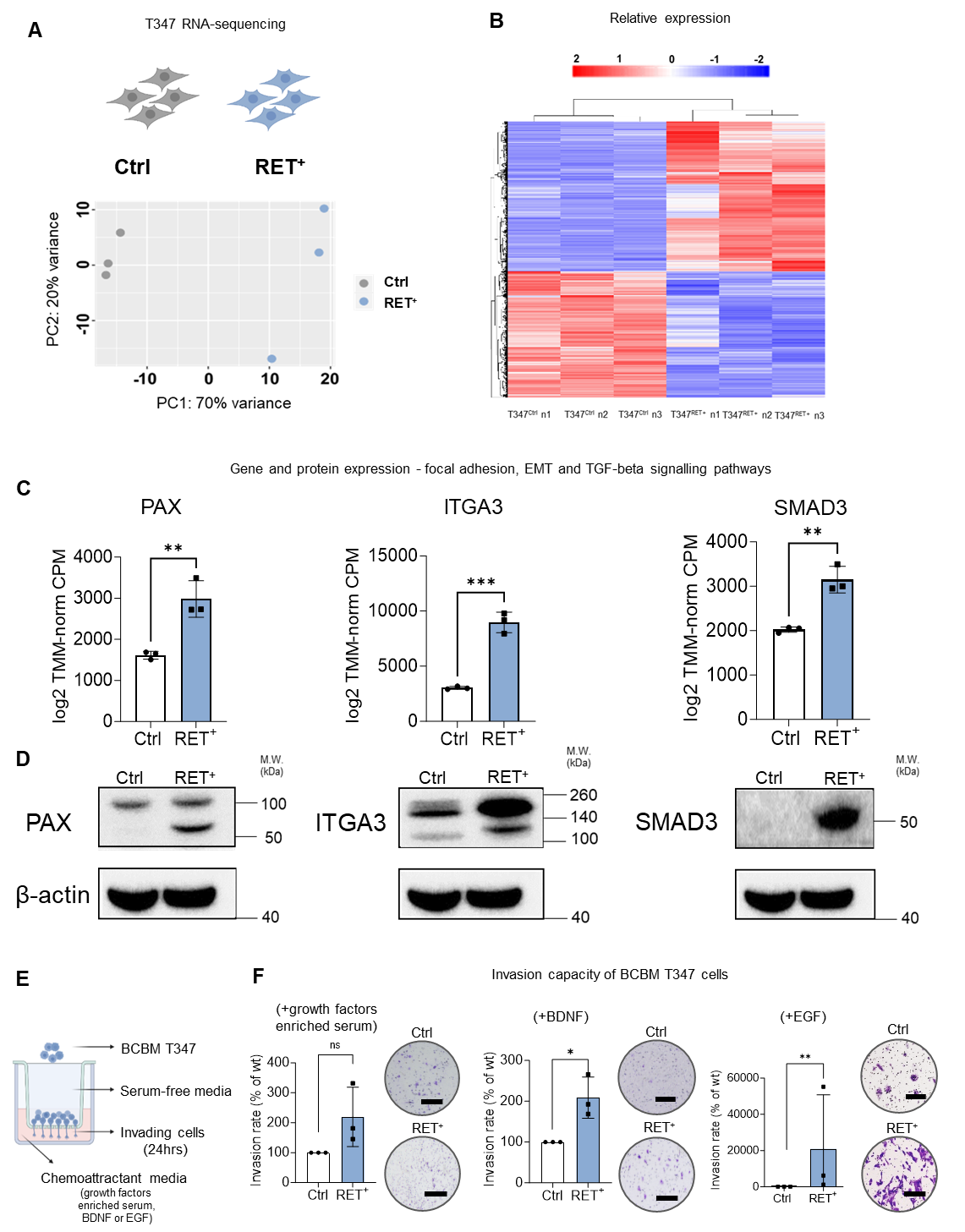


**Supplementary Figure 4. Profiling of T347-RET^+^ cells. A**. Graphical representation of RNA-sequencing performed on BCBM T347-Ctrl and T347-RET^+^ cells (top). Sample-to-sample distances visualized as principal component analysis (PCA) plot (bottom). **B**. Heatmap showing distinct gene expression patterns between T347-Ctrl and T347-RET^+^ BCBM cells. Colours indicate genes z-score (side bar). **C**. mRNA expression of Integrin Subunit Alpha 3 (*ITGA3*), Paxillin (*PAX*) and *SMAD3* in T347-Ctrl and T347-RET^+^ cells. Two-sided unpaired t-test. **, *P* < 0.01; ***, *P* < 0.001 **D**. Protein expression of PAX, ITGA3 and SMAD3 in T347-Ctrl and T347-RET^+^ cells. Representative western blot images. **E**. Graphical representation of invasion assay, done by Biorender.com. Invasion capacity was measured using a transwell assay. Growth factors enriched media (10%) FBS or BDNF (100 ng/ml) were used as chemoattractant. **F**. The invasion rate of T347-RET^+^ cells after 24 hrs compared to T347-Ctrl cells is shown on the graph (mean ± S.D. , *n* = 3 biological replicates). Two-tailed t-test. *, *P* < 0.05; **, *P* < 0.01 *P* = ns, non-significant. Representative images (scale bar 100 µm).

**
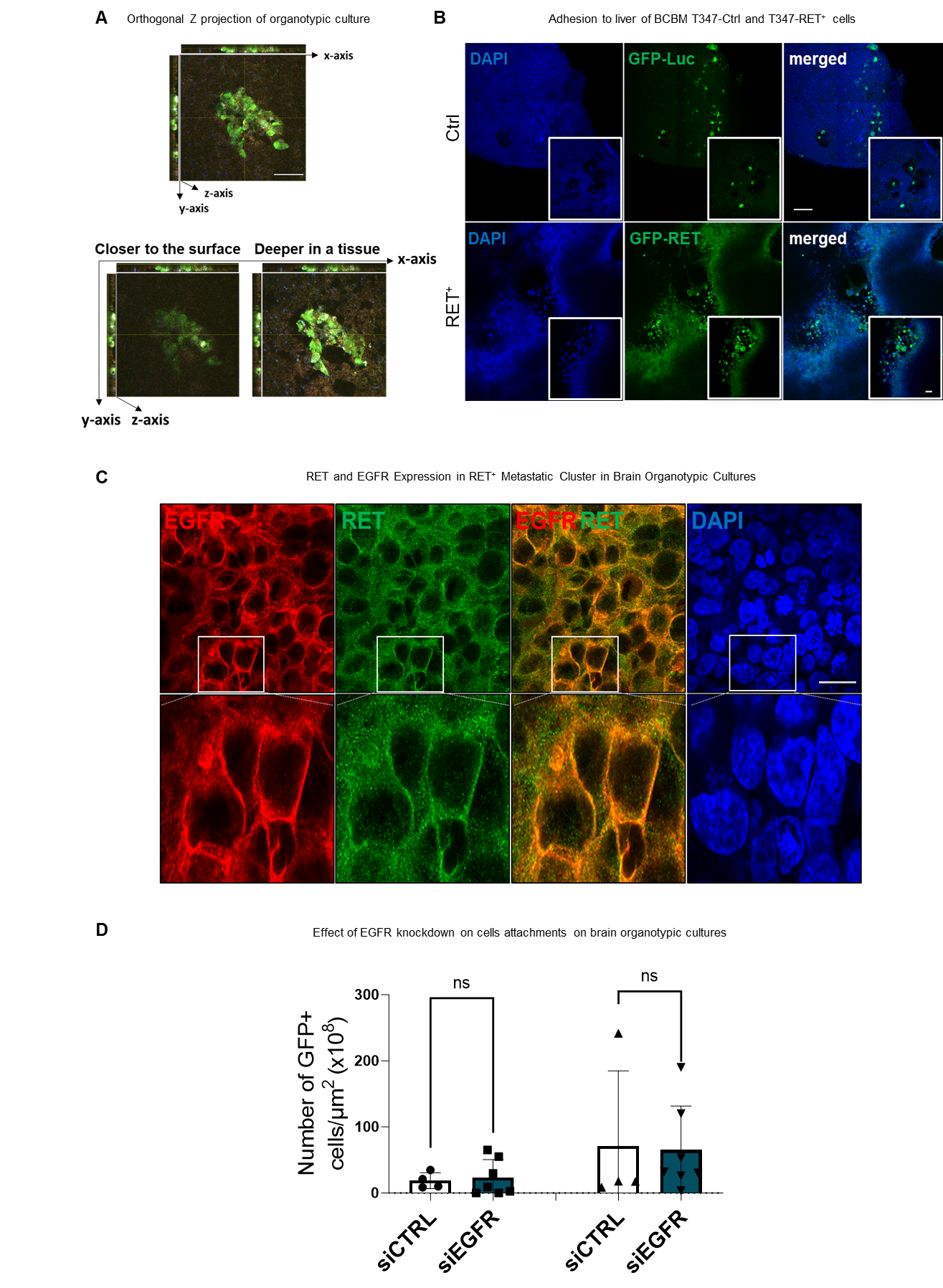
**

**Supplementary Figure 5. Characterization of T347-RET^+^ cell in the context of brain microenvironment. A.** The original stack (upper image), with orthogonal z projections of white crosshairs, is shown (14.5 μm voxel depth: 25/53 image). The lower images are examples from the z-stack showing the integration of the cells into the brain slice in brain organotypic culture (left: 4.64 μm voxel depth; right: 26.48 μm voxel depth). **B**. Representative images of BCBM T347-Ctrl and T347-RET^+^ (GFP, green; DAPI, blue) liver organotypic cultures. Original image: 10x (scale bar 200 μm); magnified overlay: 20x (scale bar 50 μm). **C**. Representative immunofluorescence images of RET (GFP, green) and EGFR (red) colocalization on brain organotypic cultures in BCBM T347-RET^+^ metastatic cluster after 72 hrs. Top images scale bar 50 μm, bottom images magnification of selected square area. **D.** Bar chart (± S.D.) shows a number of groups and single BCBM T347-RET^+^ cells attached to the brain organotypic cultures after EGFR knockdown (72 hrs, *n* = 3 biologically independent samples) normalised to the area of the brain slice. Two-sided unpaired t-test. *P* = ns, non-significant.


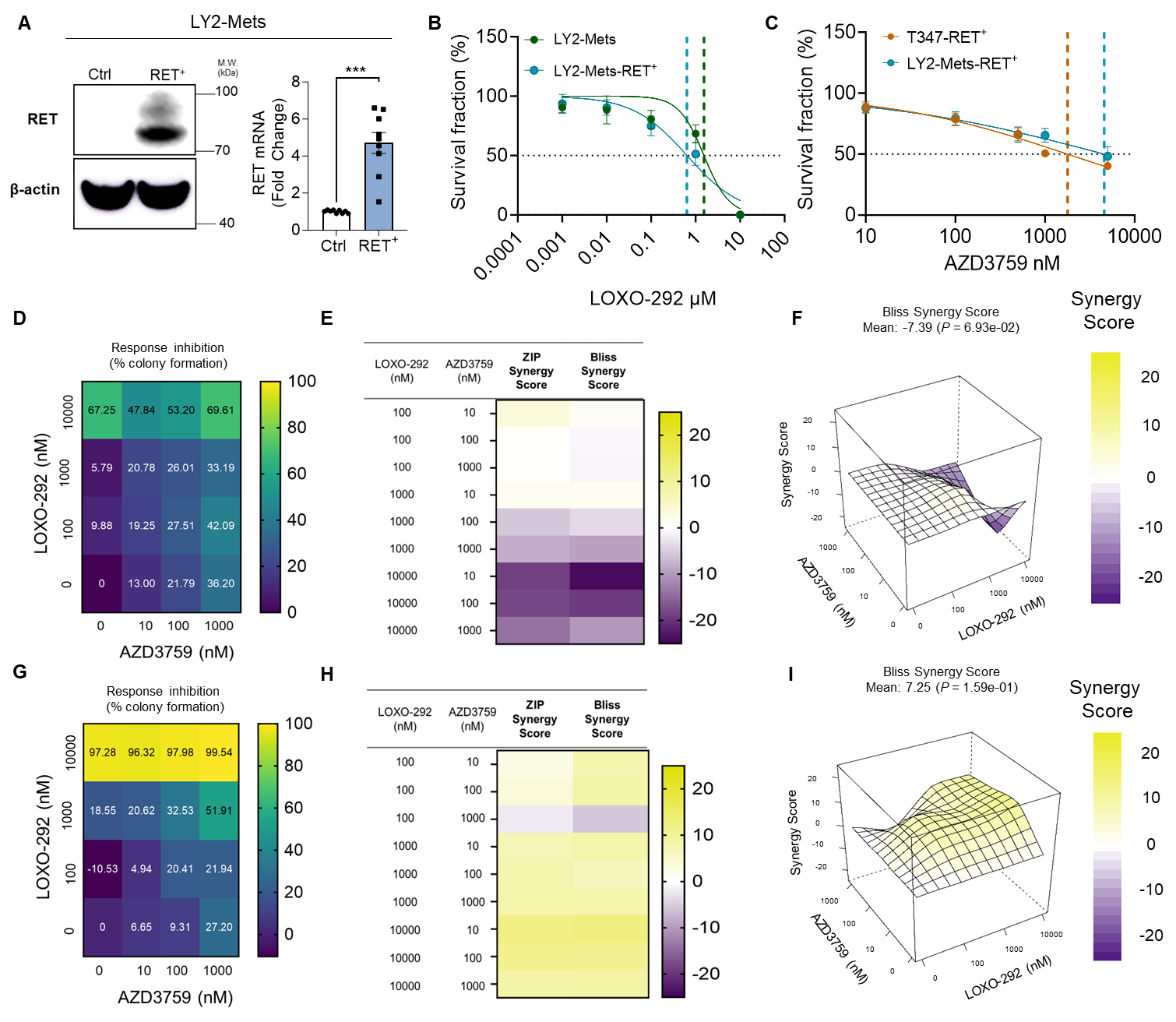


**Supplementary Figure 6**. **Effect of LOXO-292 and AZD3759 on BCBM cell colony formation**. **A**. An LY2--Mets-GFP-Luc ER+ve cells were transduced with RET RFP lentivirus and fluorescence-activated cell sorted (FACS) by flow cytometry based on RFP expression. Representative western blot of RET protein expression comparing RET expression in LY2-Mets-RET^+^ versus LY2-Mets-Ctrl cells (left) and LY2-Mets-RET^+^ mRNA gene expression (right) is shown. Bar chart (± S.D.) of mRNA *RET* expression in LY2-Mets-RET^+^ is normalized to LY2-Mets-Ctrl cells RET expression (*n* = 3). Two-sided paired t-test. ***, *P* < 0.001 **B**. Dose response of LY2-Mets and LY2-Mets-RET^+^ cell lines to LOXO-292 measured by clonogenic assay. A dotted vertical line shows half maximal inhibitory concentration (IC50, *n* = 3 biological replicates). For LY2-Mets-Ctrl IC50 value is 1.542 µM and for LY2-Mets-RET^+^ is 0.6405 µM. Data is presented as mean ± S.D. **C**. Graphical representation of AZD3759 dose-response curves in T347-RET^+^ and LY2-Mets-RET^+^ cells measured by clonogenic assay. A dotted vertical line shows half maximal inhibitory concentration (IC50) of AZD3759 for each cell line (*n* = 3-4 biological replicates). T347-RET^+^ has IC50 value of 1788 nM and LY2-Mets-RET^+^ of 4586 nM. Data is presented as mean ± S.D. **D and G**. Percentage of inhibition of colony formation upon treatment with the indicated doses of inhibitors in T347-RET^+^ (**D**) and LY2-Mets-RET^+^ (**G**) cell lines (*n* = 3-4 biological replicates). Colony formation efficacy was normalized to the DMSO-treated control. **E.** Table showing zero interaction potency (ZIP) synergy score and Bliss synergy score of the indicated LOXO-292 and AZD3759 doses combinations for T347-RET^+^. **F**. A synergistic response of the Bliss score surface in T347-RET^+^ using LOXO-292 and AZD3759 and measured by clonogenic assay. **H**. ZIP synergy score and Bliss synergy score calculated by SynergyFinder+ software of the indicated LOXO-292 and AZD3759 combinations for LY2-Mets-RET^+^ cells. **I**. 3D surface Bliss synergy score response to LOXO-292 and AZD3759 combination treatments in LY2-Mets-RET^+^.
